# Surgical Menopause and Estrogen Therapy Modulate the Gut Microbiota, Obesity Markers, and Spatial Memory in Rats

**DOI:** 10.3389/fcimb.2021.702628

**Published:** 2021-09-30

**Authors:** Lydia Zeibich, Stephanie V. Koebele, Victoria E. Bernaud, Zehra Esra Ilhan, Blake Dirks, Steven N. Northup-Smith, Rachel Neeley, Juan Maldonado, Khemlal Nirmalkar, Julia A. Files, Anita P. Mayer, Heather A. Bimonte-Nelson, Rosa Krajmalnik-Brown

**Affiliations:** ^1^ Biodesign Center for Health Through Microbiomes, Arizona State University, Tempe, AZ, United States; ^2^ Department of Psychology, Arizona State University, Tempe, AZ, United States; ^3^ Arizona Alzheimer’s Consortium, Phoenix, AZ, United States; ^4^ Genomics Core, Arizona State University, Tempe, AZ, United States; ^5^ Division of Women’s Health Internal Medicine, Mayo Clinic, Scottsdale, AZ, United States

**Keywords:** gut microbiome, gut-brain axis, ovariectomy, estrogen, memory, menopause, hormone therapy

## Abstract

Menopause in human females and subsequent ovarian hormone deficiency, particularly concerning 17β-estradiol (E2), increase the risk for metabolic dysfunctions associated with obesity, diabetes type 2, cardiovascular diseases, and dementia. Several studies indicate that these disorders are also strongly associated with compositional changes in the intestinal microbiota; however, how E2 deficiency and hormone therapy affect the gut microbial community is not well understood. Using a rat model, we aimed to evaluate how ovariectomy (OVX) and subsequent E2 administration drive changes in metabolic health and the gut microbial community, as well as potential associations with learning and memory. Findings indicated that OVX-induced ovarian hormone deficiency and E2 treatment had significant impacts on several health-affecting parameters, including (a) the abundance of some intestinal bacterial taxa (e.g., *Bifidobacteriaceae* and *Porphyromonadaceae*), (b) the abundance of microbial short-chain fatty acids (SCFAs) (e.g., isobutyrate), (c) weight/BMI, and (d) high-demand spatial working memory following surgical menopause. Furthermore, exploratory correlations among intestinal bacteria abundance, cognition, and BMI underscored the putative influence of surgical menopause and E2 administration on gut-brain interactions. Collectively, this study showed that surgical menopause is associated with physiological and behavioral changes, and that E2-linked compositional changes in the intestinal microbiota might contribute to some of its related negative health consequences. Overall, this study provides novel insights into interactions among endocrine and gastrointestinal systems in the post-menopausal life stage that collectively alter the risk for the development and progression of cardiovascular, metabolic, and dementia-related diseases.

## Introduction

From sexual maturity at puberty until reproductive senescence in mid- to late- life, female mammals experience predictable fluctuations in sex steroid hormones that control reproductive capacity (Melmed et al., 2015). Although sex steroid hormones, including estrogens, progesterone, and androgens, are primarily considered to function as the master regulators of reproduction, it has been established in the past several decades that reproductive hormones (particularly estrogens) play a key role in regulating the healthy functioning of every major body system, including the brain and gut microbiota (Rettberg et al., 2014; Kaliannan et al., 2018; Patel et al., 2018; Acharya et al., 2019). In women, unless oophorectomy (surgical intervention to remove the ovaries; in rodents, ovariectomy [OVX]) takes place earlier, menopause (a natural end in reproductive capacity in females) occurs around age 51-52 (NAMS, 2014). Menopause is defined as the permanent cessation of the menstrual cycle for more than 12 months, and ensues when the finite ovarian follicle reserve reaches a critical threshold of natural depletion (Soules et al., 2001; Hoffman et al., 2012; NAMS, 2014). Because growing ovarian follicles are the primary steroidogenesis site for estrogens and progesterone, the eventual natural exhaustion of the follicle reserve or the surgical removal of the ovaries coincides with a significant decrease in circulating ovarian hormone levels in the post-menopausal life stage. Menopause-related symptoms, including, but not limited to, changes in visceral body fat distribution, baseline body weight, metabolism, and memory function can have a significant impact on quality of life (Mitchell and Woods, 2001; Hoffman et al., 2012; Weber et al., 2013; Al-Safi and Santoro, 2014; NAMS, 2014; Leeners et al., 2017).

To alleviate physical symptoms associated with menopause, women may opt to take estrogen-containing hormone therapy (with or without a progestogen component). In particular, many popular formulations include a bioidentical analog to the most potent naturally circulating estrogen, 17β-estradiol (E2) (Kuhl, 2005; NAMS, 2014; NAMS, 2017; Files and Kling, 2020). E2 is known to have many beneficial and protective properties for the brain and body in both humans and animal models (Gibbs and Gabor, 2003; Morrison et al., 2006; Daniel and Bohacek, 2010; Koebele and Bimonte-Nelson, 2015; Luine and Frankfurt, 2020). Although hormone therapy is not currently recommended for the primary indications of metabolic dysregulation or cognitive symptoms, there is accumulating evidence in basic science research and clinical investigations that hormone therapy may attenuate menopause-associated weight gain and adipose accumulation (NAMS, 2017) as well as cognitive changes (Maki, 2006; Greendale et al., 2009; Maki et al., 2011). In addition to typical menopause symptoms, there are a number of negative health outcomes associated with a hypoestrogenic state, including increased risks of cardiovascular diseases, metabolic syndrome, obesity, diabetes, osteoporosis, sarcopenia, anxiety and depression, and neurodegenerative diseases during aging (Farrag et al., 2002; Rocca et al., 2007; Rosano et al., 2007; Vegeto et al., 2008; Rocca et al., 2012; Brand et al., 2013; Lizcano and Guzmán, 2014; Neves-e-Castro et al., 2015; Baber et al., 2016; NAMS, 2017). Given that the human lifespan is continually increasing, and women are now living up to a third of their lives in a hypoestrogenic, post-reproductive state (Sherwin, 2003; Murray et al., 2015), it is critical to better understand how menopause-associated loss of ovarian hormones, as well as treatment with exogenous estrogens and/or progestogens, can modify risk for these negative health outcomes.

The gut microbiota consists of more than 100 trillion microbes that live within the gastrointestinal tract. In addition to its role in digestion, recent studies have revealed the role of the gut microbiota in a broad array of functions related to whole-body health and function, including immune, endocrine, and brain systems (Sharon et al., 2014; Sharon et al., 2016; Rastelli et al., 2019; Rea et al., 2020). Indeed, compositional and functional changes in the gut microbiota have been implicated in numerous syndromes and disorders, comprising, but not limited to, metabolic syndrome, neurodevelopmental disorders (e.g., autism spectrum disorders), affective disorders (e.g., depression), and neurodegenerative disease states (e.g., Alzheimer’s disease and Parkinson’s disease) (Sharon et al., 2014; Dinan and Cryan, 2016; Kelly et al., 2016; Sharon et al., 2016; Kang et al., 2017; Lach et al., 2018; Tetel et al., 2018; Bastiaanssen et al., 2019; Fülling et al., 2019; Rea et al., 2020).

In this regard, altered abundances of *Firmicutes-, Bacteroidetes-, Actinobacteria-*, and *Verrucomicrobia*-related families such as *Clostridiaceae*, *Peptostreptococcaceae*, *Porphyromonadaceae, Bifidobacteriaceae, and Verrucomicrobiaceae* have been repeatedly associated with cardiovascular and neurodegenerative diseases, obesity, and type 2 diabetes (Branchereau et al., 2016; Singh et al., 2016; Jie et al., 2017; Kitai and Tang, 2017; Vogt et al., 2017; Li et al., 2018; Ticinesi et al., 2018). Likewise, compositional and abundance changes in fermentation derived short-chain fatty acids (SCFAs) like butyrate and branched SCFAs like isobutyrate have a role in fulfilling energy requirements of the host, but also have the potential to modulate aspects of human metabolism (e.g., through their alterations of fatty acid oxidation and lipolysis or neuroactive properties (Van Treuren and Dodd, 2020). Many syndromes and disorders associated with gut dysbiosis co-occur with menopause and estrogen deficiency, pointing to a potential interaction among systems.

Given that there are many direct connections between the gut-brain axis, including the enteric nervous system and the vagus nerve (Goehler et al., 2000; Fülling et al., 2019) it is reasonable to deduce that the gut microbiota and the central nervous system have substantial interplay with one another to influence the development and progression of disease beyond simple epiphenomenon (Zapata and Quagliarello, 2015; Dinan and Cryan, 2016; Sharon et al., 2016; Tetel et al., 2018). Furthermore, gut microbiota play a significant role in endocrine regulation, including key conjugation reactions in the gut that result in the regulation of bioavailability of hormone molecules, including sex steroid hormones (Baker et al., 2017; Ervin et al., 2019). Indeed, this relationship is bidirectional; estrogen is known to modulate the gut microbiota (Kaliannan et al., 2018; Acharya et al., 2019), and in turn a specific subset of gut microbiota, the estrobolome, modulates the bioactivity of estrogen (Kwa et al., 2016) by producing enzymes such as *beta*-glucuronidases and *beta*-glucosidases which de-conjugate and reactivate E2 (Baker et al., 2017; Tetel et al., 2018). Although aging, hormone status, and cognitive outcomes have each been linked to gut microbiota changes (Rogers et al., 2009; O’Toole and Jeffery, 2015; Zapata and Quagliarello, 2015; Dinan and Cryan, 2016; Leeners et al., 2017; Iwasa et al., 2018), until now, little research has been dedicated to understanding the intersection between ovarian hormones, menopause-related ovarian hormone decline, gut microbiota, and behavior. Despite the dearth of work in this area, it is clear that there are important interactions among these factors and this gap in the literature should be further explored (Tetel et al., 2018). Furthermore, recently published rodent-based studies have demonstrated compositional changes in the intestinal microbiota after E2 treatment - including altered abundances of *Bacteroidetes, Firmicutes, Actinobacteria, and Proteobacteria* (Kaliannan et al., 2018; Acharya et al., 2019), indicating a putative association between ovarian hormones and gut microbial composition. Whether altered function and composition within the gut microbiota initiates these changes, or whether erratic ovarian hormone levels observed during the menopause transition trigger gut dysbiosis, remains to be discovered. Regardless of the directionality of the effect, these factors work in concert to increase the risk for metabolic syndrome and other negative health outcomes in the post-menopausal state and require further investigation.

Here, we evaluated the impact of OVX and subsequent daily E2 treatment on gut microbiota composition and function, obesity markers, and cognition in middle-aged F344-CDF female rats. Following OVX or Sham-control surgery, rats were administered a vehicle control (sesame oil) or daily treatment with a low or high dose of E2 (**Table 1**) to methodically assess the role of E2 administration and dose on regulating gut microbiota composition and function after OVX as well as compared to ovary-intact Sham rats. In addition to profiling gut microbiota composition and fatty acids production following OVX and subsequent E2 treatment, rats were tested on a complex spatial working memory task, allowing for investigation into the role of surgical menopause and E2 administration on learning and memory, as well as an exploration of potential relationships among behavioral outcomes and gut microbiota. This research provides a comprehensive analysis of the gut microbiota, obesity-related health factors, and behavioral effects following surgical menopause with or without E2 treatment, and offers insights into potential mechanisms underlying estrogen-gut-brain interactions during aging and the menopausal state.

**Table 1:** Experimental group condition descriptions and treatments.

Group	Ovary/menopause status	Hormone administration	Analog to human state
**Sham-Vehicle**	Ovary-intact (control)	Control injection (0.1 mL sesame oil) daily	Ovary-intact aging female with no hormone therapy
**OVX-Vehicle**	Ovaries surgically removed	Control injection (0.1 mL sesame oil) daily	Surgically menopausal female with no hormone therapy
**OVX-E2-Low**	Ovaries surgically removed	2 µg E2 dissolved in 0.1 mL sesame oil daily	Surgically menopausal female with low-dose E2 hormone therapy
**OVX-E2-High**	Ovaries surgically removed	4 µg E2 dissolved in 0.1 mL sesame oil daily	Surgically menopausal female with high-dose E2 hormone therapy

## Materials and Methods

### Subjects, Surgery, and Hormone Treatment

Forty female Fischer-344-CDF (F344) rats without sexual experience were obtained from the National Institute on Aging CRL colony (Raleigh, NC). Rats were 11 months of age upon arrival to the Arizona State University (ASU) Department of Psychology vivarium. Rats were pair-housed, maintained on a 12-hour light/dark cycle (7 am lights on/7 pm lights off) and had free access to Teklad Global 18% Protein Rodent Diet chow (#2018, Envigo) and water for the entirety of the experiment. Subjects were given one week to acclimate to the vivarium prior to beginning experimental procedures (for detailed experimental timeline, see **Supplemental Figure S1**). All procedures were approved by the ASU Institutional Animal Care and Use Committee and adhered to standards set forth by the National Institutes of Health.

One week after arrival, rats were randomly assigned to undergo OVX (n=30) or Sham (n=10) surgery. All rats were anesthetized using inhaled isoflurane anesthesia. Rats assigned to the OVX surgery condition received bilateral dorsolateral incisions through the skin, subcutaneous fat, and muscle to expose the peritoneal cavity. Ovaries were separated from the visceral fat, ligated, and removed. The muscle incision was closed using dissolvable Vicryl suture. Bupivacaine (0.25%; Marcaine Pfizer Pharmaceutical, Hospira Inc., Lake Forest, IL) was applied directly on the muscle incision prior to surgical stapling of the skin incision. Rats assigned to the Sham surgery condition received bilateral dorsolateral skin incisions (~1cm) only. Bupivacaine (0.25%; Marcaine; Pfizer Pharmaceutical, Hospira Inc., Lake Forest, IL) was applied topically on the muscle, and then the skin incisions were closed with surgical staples. All rats received 5 mg/kg carprofen (Rimadyl) and 2 mL sterile saline to prevent postsurgical dehydration.

Forty-eight hours after Sham or OVX surgery, rats were re-housed with their prior cage mate. One rat was single-housed for the remainder of the experiment due to cage mate death after surgery. OVX rats were then assigned to one of the following treatment groups: OVX-Vehicle (n = 10; 0.1 mL sesame oil), OVX-E2 Low (n = 10; 2 µg E2/day in 0.1 mL sesame oil; E2: #E8875, Sigma-Aldrich, St. Louis, MO), or OVX-E2 High (n = 9; 4 µg E2/day in 0.1 mL sesame oil). Rats in the Sham group received a daily control Vehicle injection (n = 10; 0.1 mL sesame oil). Pair-housed rats within a cage were always assigned to the same treatment group. **Table 1** provides a summary of surgery and treatment group assignments. Daily subcutaneous injections began 48 hours after OVX or Sham surgery (i.e., the day rats were re-pair-housed after surgery), and injections were administered daily in the morning for the duration of the experiment until euthanasia (23 days total; **Figure S1**).

### Obesity-Related Factors: Body Weight and Girth Measurements

Beginning at surgery, body weight (g) was recorded daily for the entirety of the experiment. At surgery, while rats were anesthetized, body length (cm) from snout to rump (minus the tail) was measured and recorded. To obtain measures of girth, thoracic circumference (cm) was obtained using a flexible measuring tape immediately posterior to the forelimbs, and abdominal circumference (cm) was obtained at the widest point of the abdomen anterior to the hindlimbs (Novelli et al., 2007). At the end of the experiment, following euthanasia, thoracic and abdominal circumference were measured again to determine the change across time with Sham or OVX surgery and any subsequent E2 treatment.

### Vaginal Smears

Four days after injections began, estrous cycle vaginal cytology monitoring was completed for three consecutive days. A small cotton-tipped applicator was dipped in sterile saline and gently inserted into the vaginal canal to collect cells. Cells were observed under a light microscope at 10x magnification and classified as either proestrus, estrus, metestrus, or diestrus phases of the estrous cycle, as previously published (Goldman et al., 2007; Koebele and Bimonte-Nelson, 2016).

### Behavior Testing: Water Radial-Arm Maze and Visible Platform

Ten days after the onset of daily E2 or Vehicle-control treatment administration, rats were evaluated on the spatial working and reference memory water escape task, the water radial-arm maze (WRAM; Bimonte and Denenberg, 1999; Bimonte et al., 2000; Bimonte et al., 2002; Bimonte et al., 2003; Bimonte-Nelson et al., 2003a; Bimonte-Nelson et al., 2004; Bimonte-Nelson et al., 2015). Details regarding the WRAM apparatus and experimental testing procedures were identical to previously published work (for example, see: Bimonte-Nelson et al., 2015; Prakapenka et al., 2018; Koebele et al., 2020a; Koebele et al., 2020b). Briefly, WRAM testing consisted of four trials per day, one for each platform, for 13 consecutive days. Day one was considered maze procedure acquisition, days 2-12 were normal baseline testing days, and a delayed memory retention test was implemented on day 13. As the number of platform locations to be recalled increased with each trial, working memory load became gradually challenged across each daily testing session, such that trials three and four were considered high working memory load trials, with trial four designated as the maximum working memory load trial. On Day 13, a four-hour delay between trials two and three was employed to assess memory retention over a delayed interval. Performance on the WRAM was quantified by calculating the total number of non-platformed arm entries rats made on each trial prior to locating an escape platform, as previously published (Bimonte-Nelson et al., 2015). One day after the WRAM delay test, rats were evaluated on a Visible Platform control task to assess visual and motor acuity, both of which are required to effectively solve water-escape based maze tasks. The apparatus and testing procedures were identical to previously published protocols (e.g., Bimonte-Nelson et al., 2015; Prakapenka et al., 2018; Koebele et al., 2020a; Koebele et al., 2020b). Performance was assessed by recording latency (s) to the visible platform on each trial.

### Euthanasia and Sample Extraction

Rats were approximately 12 months of age at the end of the experiment. One day after the Visible Platform task, rats were euthanized. Body weight was recorded prior to euthanasia. Rats were deeply anesthetized with inhaled isoflurane anesthesia prior to decapitation. Thoracic and abdominal circumference were measured on the body using a flexible tape measure. Then, uteri were dissected from the body cavity, cleared of excess fat, and weighed immediately. The large intestine and colon were separated from the surrounding visceral adipose tissue and dissected from the abdominal cavity by making one cut at the pyloric sphincter and a second cut at the rectum. A fecal sample and a tissue sample from the area surrounding the bolus was collected from the proximal and distal colon with sterile instruments (**Supplemental Figure S2**). Tissue samples were rinsed with PBS to clear any remaining fecal matter from the sample. Fecal and tissue samples were then placed in 1.5 mL microcentrifuge tubes and stored at -70°C until analysis.

### Chemical and Molecular Analyses

High performance liquid chromatography (HPLC; Shimadzu, Kyoto, Japan) was used to quantify SCFAs in fecal samples. The organic acids were separated and identified by using a Bio-Rad column (Aminex HPX-87H) at 65°C, 5 mM H_2_SO_4_ as mobile phase, and a 0.6 mL/min flow rate that was increased to 0.8 mL/min at 30 minutes.

DNA extraction, 16S rRNA gene amplicon sequencing using the illumina platform and sequence analysis with Qiime2 (v2020.2) was conducted as described before (Bolyen et al., 2019). Sequence quality control was performed with DADA2 (Callahan et al., 2016). Based on the unsatisfactory quality of the reverse reads, which would prevent a sufficient merging, only the forward reads (250 bp) were used throughout the sequence analysis. The rarefaction analyses revealed that most of the phylotypes were targeted (**Supplemental Figure S3**), indicating a reliable representation of the community diversity. Seven single samples were eliminated from the sequence analysis since these samples had less than 13,000 reads and thus a compromised phylotype assignment-quality and quantity (**Supplemental Figure S3**). Taxonomic assignment was realized with Greengenes database (v13.8) considering 99% OTU similarities (McDonald et al., 2012). The taxonomic identity of the closest related species of a significant phylotype was determined by BLAST (Basic Local Alignment Search Tool) using the currently available database at NCBI (National Center for Biotechnology Information). Alpha diversity analysis was calculated with Faith’s PD (Faith, 1992). Beta diversity analysis was calculated with both weighted and unweighted Unifrac distance metrics (Lozupone et al., 2006).

Phylogenetic Investigation of Communities by Reconstruction of Unobserved States (PICRUSt2, v2.3.0-b) was used to link 16S rRNA gene amplicon sequences to known reference genomes to predict gene family abundances in host-associated microbial communities (Langille et al., 2013; Douglas et al., 2020). Thus, this bioinformatics software package enabled the prediction of functional properties of the gut microbial communities of the rats used.

### Statistical Analyses

Linear discriminant analysis effect size (LEfSe) (Segata et al., 2011) was used to identify significant differences between the microbial community composition (16S rRNA Genes) and function (SCFAs and predicted pathways) of OVX-Vehicle and OVX-E2-High rats. The same analysis was conducted to identify significant compositional differences between Sham-Vehicle rats and OVX-Vehicle, OVX-E2-Low, or OVX-E2-High rats. Univariate analyses (t-test, Mann-Whitney *U* test etc.) were performed based on the distribution of the data/normality test. All p values were corrected for multiple testing using the Benjamini-Hochberg False Discovery Rate method and assigned as *q* value. P and *q* values were calculated in R and values ≤ 0.05 were considered as statistically significant.

Behavioral data analyses were processed using Statview statistical software. The independent variable for all two-group comparisons was Treatment (Sham-Vehicle, OVX-Vehicle, OVX-E2-Low, OVX-E2-High). WRAM data were assessed using repeated measures ANOVA, with Total Errors as the dependent variable, and Trials nested within Days as the repeated measures. Day 1 was considered task acquisition and was not included in the analyses. Based on previous findings from our laboratory, WRAM data were blocked into the Learning Phase (Days 2-8) and the Asymptotic Phase (Days 9-12), as published previously (Bimonte and Denenberg, 1999; Bimonte-Nelson et al., 2003a; Bimonte-Nelson et al., 2003a; Bimonte-Nelson et al., 2004; Mennenga et al., 2015a; Braden et al., 2017; Koebele et al., 2020a; Bernaud et al., 2021). *A priori* planned analyses evaluating high working memory load trials (Trial 3 and Trial 4) were also completed, as our laboratory has previously reported age- and hormone- mediated effects when working memory load is sufficiently burdened (Bimonte and Denenberg, 1999; Mennenga et al., 2015a; Mennenga et al., 2015b; Koebele et al., 2019; Koebele et al., 2020b). Delay data were evaluated by comparing total error scores for Trial 3 during the last session of baseline testing compared to total error scores for Trial 3 (the first post-delay trial) on Day 13 for each treatment group. Visible Platform performance was analyzed using repeated measures ANOVA for each treatment group with Latency to Platform (s) as the dependent variable and Trial 1 *vs* Trial 6 as the repeated measure.

Daily body weight data were analyzed using repeated measures ANOVA to investigate the trajectory of change across time. Obesity markers, including Abdominal Circumference, Thoracic Circumference, Body Weight, and BMI at the beginning and end of the experiment were analyzed using ANOVA, with Treatment as the independent variable and Abdominal Circumference (cm), Thoracic Circumference (cm), Body Weight (g), and BMI as the dependent variables, respectively. A separate ANOVA for net change in each of these obesity markers across the experiment was also completed. Uterine weight data (g) obtained at the end of the experiment were also evaluated using ANOVA, with Treatment as the independent variable and wet uterine weight as the dependent variable. All two-group ANOVA and repeated measures ANOVA analyses were two-tailed with an α level set to 0.05. When applicable, Fisher’s PLSD *post hoc* results are presented. Results from the data analyses were considered marginal when the p value fell between 0.05 and 0.10.

Spearman ρ correlation analysis was performed by using the software jmp from SAS (Cary, NC, USA). For correlation analyses, BMI at the end of the experiment was calculated using weight (g)/body length (cm)^2^ for each rat. Total Max Load (TML) memory scores for the Learning Phase and Asymptotic Phase of the WRAM were calculated by averaging the total number of errors committed on Trial 4, the maximum working memory load trial, for each rat. The resulting value for each block of testing was used as a representation of maximally burdened working memory load to correlate with gut microbial data. Spearman’s ρ correlations were completed between microbial data and BMI, and microbial data and TML scores for each treatment group. Unless otherwise noted, Sham-Vehicle n = 10, OVX-Vehicle n = 10, OVX-E2-Low n = 10, and OVX-E2-High n = 9 for all analyses.

### Accession Numbers

Sequences were deposited at the European Nucleotide Archive (ENA) under study number PRJEB40917. Representative sequences of phylotypes with ≥0.1% relative abundance were deposited under the accession numbers LR898068 to LR898243.

## Results

### Surgical Menopause and Estrogen Therapy Modulate Gut Microbiota Composition and Function

E2-linked effects on the overall intestinal microbial community composition were assessed by 16S rRNA gene amplicon sequencing analyses. A total of 7,200,208 bacterial 16S rRNA gene amplicon sequences were obtained. Alpha diversity analysis based on Faith Diversity Index (Faith, 1992) indicated no significant differences within Sham-Vehicle, OVX-Vehicle, OVX-E2-Low, and OVX-E2-High rats when distal and proximal or mucosal and fecal samples were merged (boxplots; **Supplemental Figure S4A**). However, separate sample analysis showed a sigificant difference between proximal samples of Sham-Vehicle and OVX-Vehicle. Further analysis, irrespective of the different treatment groups, indicated significantly higher microbial diversity in (a) mucosal samples than in fecal samples and (b) in distal colon samples than in proximal colon samples (**Supplemental Figure S4B**). Weighted UniFrac analysis displayed no clear compositional differences in fecal, mucosal, distal, and proximal samples or differences of either Sham-Vehicle, OVX-Vehicle, OVX-E2-Low, and OVX-E2-High rats (**Supplemental Figures S5A**, **B**). There is a grouping in unweighted UniFrac analysis (**Supplemental Figure S6**) wherein mucosal samples separate from fecal samples. Unweighted UniFrac analysis provides information about changes in the less abundant taxa, indicating that minor taxa were different in mucosal samples than in fecal samples (Tang et al., 2015). Unknown factors may have contributed to the clustering illustrated in **Figure S6**, a matter that could be addressed in future studies.

At the phylum level, we found 17 phyla (including candidate phyla). The fecal and mucosal gut microbiota was dominated by 54.91% *Firmicutes* (51.62% *Clostridiales* and 1.52% *Turicibacterales*), 32.91% *Bacteroidetes* (33.90% *Bacteroidales*), and 7.70% *Verrucomicrobia* (7.70% *Verrucomicrobiales*) (**Supplemental Figure S7**).

**Figure 1:**
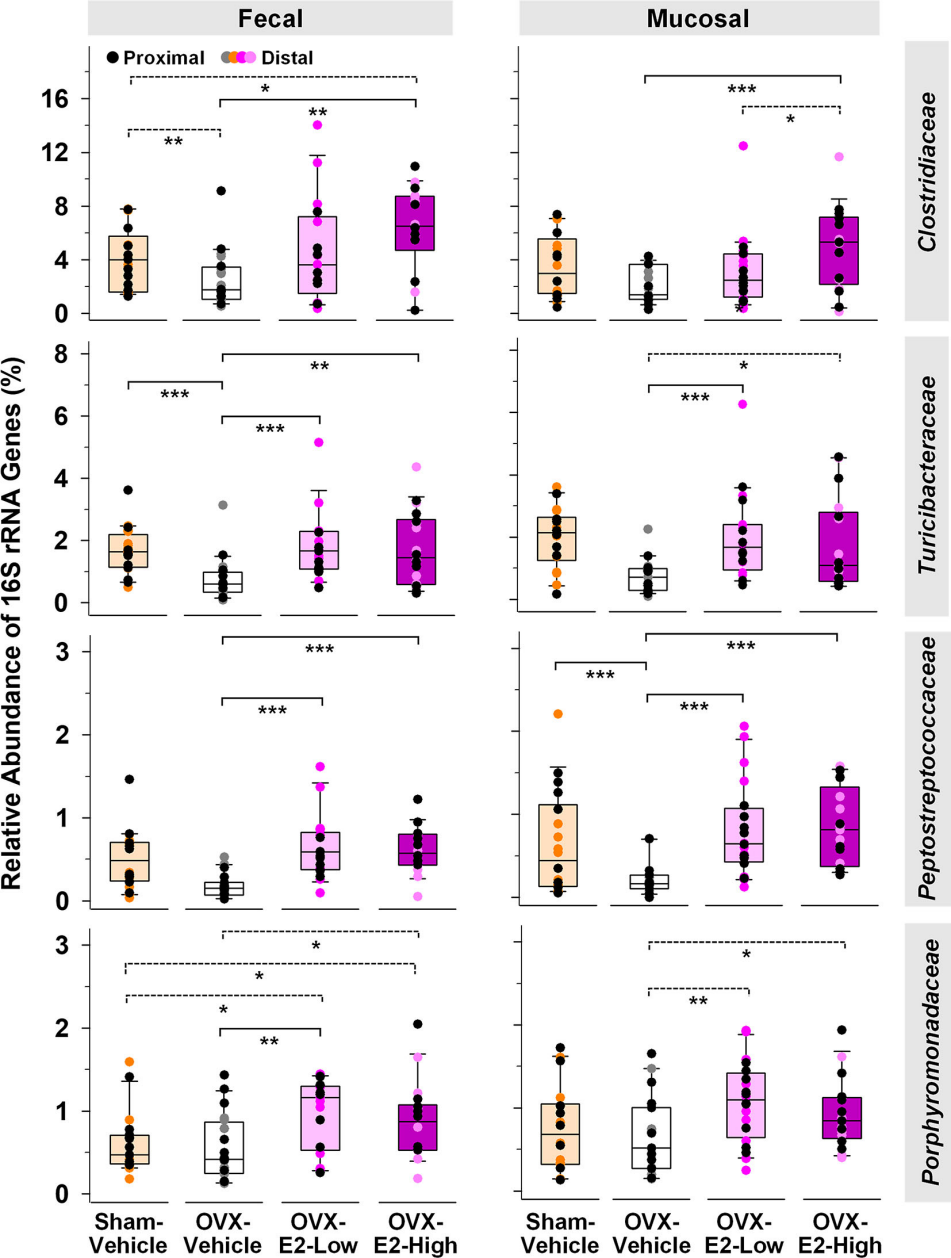
Microbial gut phylotypes at the family level that displayed significantly higher (Linear discriminant analysis effect size [LEfSe]) relative abundances in OVX-E2-High rats than in OVX-Vehicle rats. Statistical analysis was limited to families that had a ≥ 2% relative abundance in at least one sample. The asterisks indicate significant differences (*p ≤ 0.05; **p ≤ 0.01; ***p ≤ 0.001). Solid lines between groups indicate significant q values. Dashed lines between groups indicate non-significant q values (q ≥ 0.05). Group abbreviations (e.g., Sham-Vehicle) are described in **Table 1**. Error bars indicate standard deviations.

Abundance of phylotypes associated with *Clostridiaceae* (*Firmicutes*), *Turicibacteraceae* (*Firmicutes*), *Peptostreptococcaceae* (*Firmicutes*), and *Porphyromonadaceae* (*Bacteroidetes*) were significantly higher in OVX-E2-High rats than in OVX-Vehicle rats, independent of the type of sample (fecal or mucosal) (**Figure 1**). The relative abundance of fecal *Clostridiaceae* and *Turicibacteraceae*, and mucosal *Peptostreptococcaceae* in OVX-Vehicle rats was significantly lower than in Sham-Vehicle rats. At the species level, phylotype PT3 (99% sequence identity to *Clostridium saudiense*, *Clostridiaceae*), phylotype PT7 (97% sequence identity to *Turicibacter sanguinis*, *Turicibacteraceae*), phylotype PT50 (100% sequence identity to *Romboutsia timonensis*, *Peptostreptococcaceae*), and phylotype PT13 (100% sequence identity to *Parabacteroides goldsteinii*, *Porphyromonadaceae*) showed a significantly higher relative abundance in OVX-E2-High rats in comparison to OVX-Vehicle rats (**Supplemental Figure S8A**). Similar to the family-level analysis, the relative abundance of phylotype PT3 (closely related to *C. saudiense*), phylotype PT7 (closely related to *T. sanguinis*), and phylotype PT50 (closely related to *R. timonensis*) in OVX-Vehicle rats was significantly lower than in Sham-Vehicle rats. In contrast, phylotype PT12 closely related to cultured members of *Kineothrix* (*Lachnospiraceae*), phylotype PT18 closely related to cultured members of *Ruminococcus* (*Ruminococcaceae*), and phylotype PT21 closely related to cultured members of *Muribaculum* (*Muribaculaceae*), displayed significantly lower relative abundances in OVX-E2-High rats in comparison to OVX-Vehicle rats (**Supplemental Figure S8B**), suggesting that these taxa were likely (a) inhibited by E2 or (b) replaced by aforementioned microbes that displayed increased abundances in Sham-Vehicle, OVX-E2-Low, and OVX-E2-High rats compared to OVX-Vehicle rats (**Figure 1** and **Supplemental Figure S8B**).

The *Verrucomicrobiaceae*-related species *Akkermansia muciniphila* is well known to be negatively correlated with obesity and other health disorders (Depommier et al., 2019; Xu et al., 2020). Interestingly, *Verrucomicrobiaceae* were significantly lower in OVX-E2-High rats compared to OVX-Vehicle rats when fecal and mucosal samples were combined for statistical analysis (LEfSe; Kruskal–Wallis p value = 0.043). However, a separate analysis of fecal and mucosal samples clearly illustrates in mucosal samples (a) a gradual lower relative abundance of *Verrucomicrobiaceae* with High-E2 compared to Low-E2 doses and Vehicle treatment in OVX rats and (b) a significantly lower relative abundance of this family in fecal samples of Sham-Vehicle rats compared to OVX-Vehicle, OVX-E2-Low, or OVX-E2-High rats (**Supplemental Figure S9**). The relative abundance of the most dominant phylotype of this common intestinal family, phylotype PT1 - closely related to *A. muciniphila* (100% sequence identity), did not seem significantly affected by the E2 treatments (OVX-Vehicle compared to OVX-E2-High; **Supplemental Figure S9**). Nonetheless, as observed at the family-level, the relative abundance of phylotype PT1 (closely related to *A. muciniphila*) was gradually lower from no E2 (OVX-Vehicle) treatment to a High-E2 dose after OVX (**Supplemental Figure S9**), a finding more obvious in mucosal samples than in fecal samples. Furthermore, the highest relative abundance of phylotype PT1 (closely related to *A. muciniphila*) in this study was observed in OVX-Vehicle rats whereas the lowest relative abundance of this phylotype was associated with intact (Sham-Vehicle) rats (**Supplemental Figure S9**). Despite E2 treatment, this phylotype was significantly more abundant in (a) fecal samples when Sham-Vehicle rats were compared with OVX-E2-Low and (b) mucosal samples when Sham-Vehicle rats were compared with OVX-E2-Low rats (**Supplemental Figure S9**).

**Figure 2:**
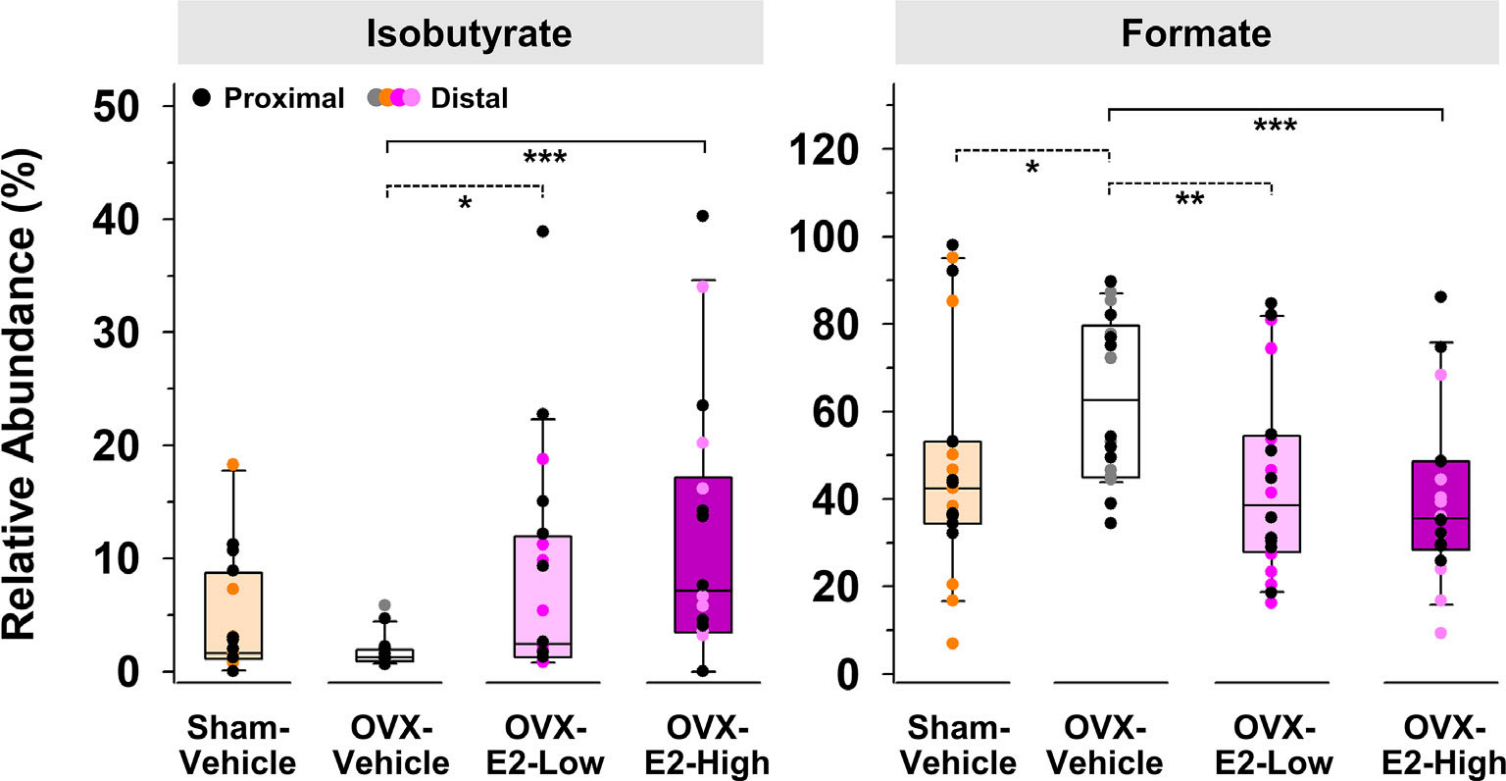
Short-chain fatty acids that displayed a significantly (LEfSe) higher or lower relative abundance in OVX-E2-Low or OVX-E2-High rats compared to OVX-Vehicle rats. The asterisks indicate statistically significant differences (*p ≤ 0.05; **p ≤ 0.01; ***p ≤ 0.001). Solid lines between groups indicate significant q values (q ≤ 0.05). Dashed lines between groups indicate non-significant q values. Group abbreviations (e.g., Sham-Vehicle) are described in **Table 1**. Error bars indicate standard deviations.

Because of large fluctuations in total concentrations of SCFAs observed from individuals within the same group (**Supplemental Tables S1** and **S2**), most likely caused by re-ingestion of feces within a pair-housed cage (Bogatyrev et al., 2020), relative abundances of produced SCFAs were used to identify E2-linked differences between the treatment groups. The relative abundance of isobutyrate was significantly enhanced in gut contents obtained from OVX-E2-High rats and OVX-E2-Low rats compared to that obtained from OVX-Vehicle rats (**Figure 2**). Additionally, the abundance of isobutyrate seemed to have a dose-dependent response, with gradually higher abundance concomitant to an increase of E2 treatment after OVX (**Figure 2**). In marked contrast, the relative abundance of formate was significantly higher in OVX-Vehicle rats compared to all other groups (**Figure 2**).

PICRUSt analysis was used to predict functional differences of microbial gut communities described above. In mucosal samples, predicted *beta*-glucuronidase was significantly less abundant in OVX-E2-High rats than in Sham-Vehicle, OVX-E2-Low, and OVX-Vehicle rats (**Supplemental Figure S10**), suggesting a negative effect of high dose E2 on microbes that harbor this specific enzyme. Interestingly, OVX itself - without subsequent E2 administration (OVX-Vehicle) - did not significantly affect the predicted abundances of *beta*-glucuronidase or *beta*-glucosidase in comparison to Sham-Vehicle.

**Figure 3:**
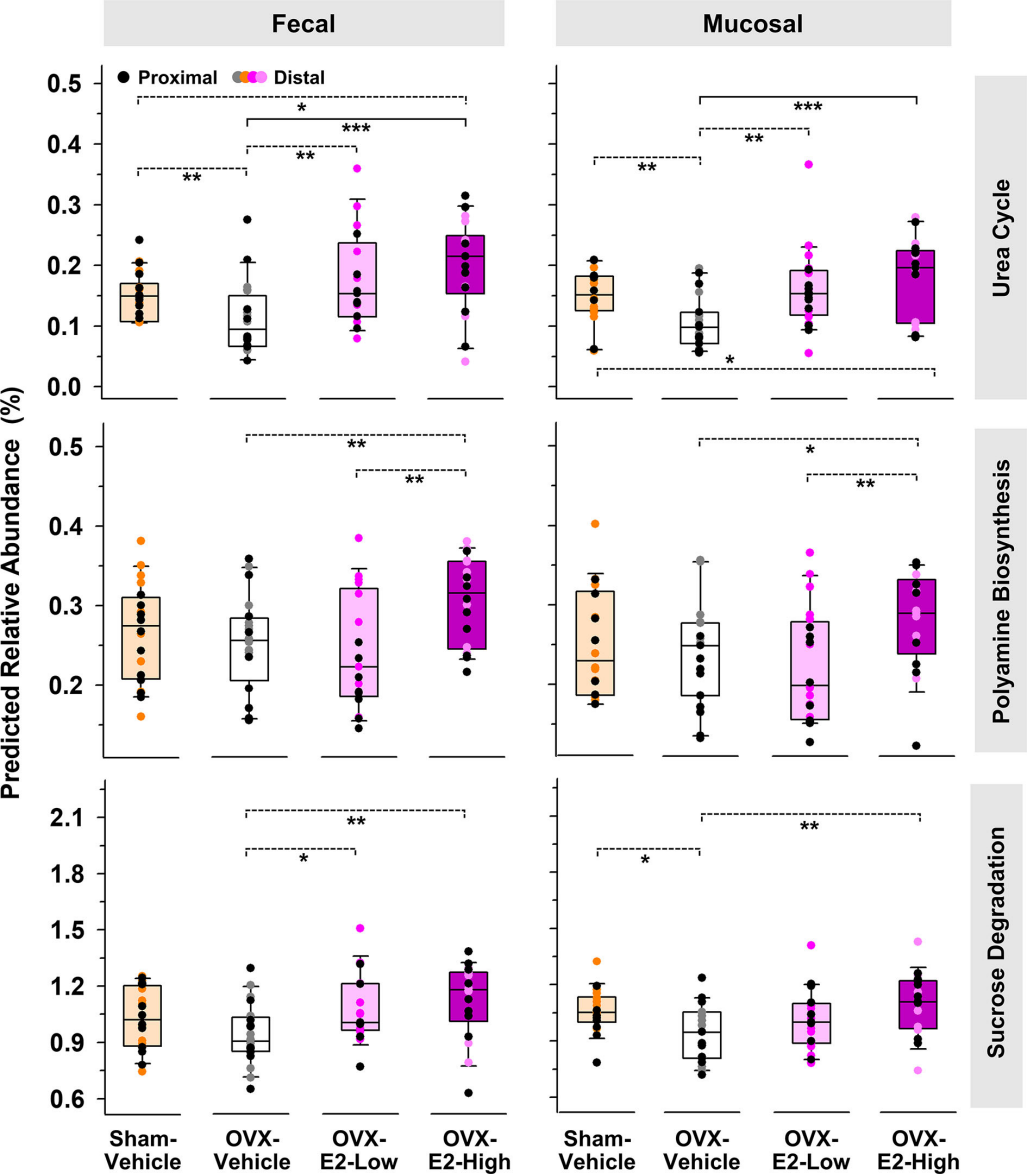
Predicted pathways (based on 16S rRNA gene sequences) that displayed a significantly (LEfSe) higher relative abundances in OVX-E2-High rats than in OVX-Vehicle rats. Relative abundance of polyamine biosynthesis and sucrose degradation represents the sum of the predicted relative abundance of arginine and polyamine biosynthesis and polyamine biosynthesis I and sucrose degradation III and IV, respectively (**Supplemental Figure S9**). Urea cycle: PWY-4984. The asterisks indicate significant differences (*p ≤ 0.05; **p ≤ 0.01; ***p ≤ 0.001). Solid lines between groups indicate significant q values (q ≤ 0.05). Dashed lines between groups indicate non-significant q values. Group abbreviations (e.g., Sham-Vehicle) are described in **Table 1**. Error bars indicate standard deviations.

The same predictive analysis indicated that the relative abundance of 16S rRNA gene sequences closely related to sequences of microbes that harbor at least one gene ortholog required for an intact urea cycle (PWY-4984) were significantly higher in the intestinal microbiota of OVX-E2-High rats compared to OVX-Vehicle rats (**Figure 3**). Furthermore, microbes that potentially harbor gene orthologs needed for the production of polyamines (e.g., putrescine) were significantly more abundant in OVX-E2-High rats compared to OVX-Vehicle rats (**Figure 3** and **Supplemental Figure S11A**). Additionally, this analysis revealed a predicted higher abundance of gene orthologs for sucrose degradation concomitant with an increase of administered E2 dose among treatment groups (**Figure 3** and **Supplemental Figure S11B**). The relative abundance of aforementioned predicted pathways was lower after OVX (Sham-Vehicle compared to OVX-Vehicle) – an observation more obvious in fecal samples. The collective findings suggest that OVX has a negative effect on these predicted pathways, but this can be compensated for with E2 administration. In this regard, rats that were treated with the lower E2 dose were most similar to rats with intact ovaries.

Besides E2 production, the surgical removal of the ovaries also affects circulating levels of other hormones such as progesterone, androgens, follicle stimulating hormone, and luteinizing hormone (Koebele and Bimonte-Nelson, 2016). Using LEfSe, we investigated the gut microbial community differences between rats with intact ovaries and OVX rats with and without estrogen treatment. Based on relative abundances, especially *Bifidobacteriaceae and Turicibacteraceae* were significantly associated with the Sham-Vehicle rats (**Table 2**), indicating a significantly lower relative abundance of these families in OVX rats, independent of subsequent E2 administration. Interestingly, *Bifidobacteriaceae* dominated by phylotype PT33 (100% sequence identity to *Bifidobacterium longum*) *and Erysipelotrichaceae*, dominated by phylotype PT69 (99% sequence identity to *Clostridium cocleatum*), were significantly higher in fecal samples of Sham-Vehicle rats compared to OVX-E2-Low and OVX-E2-High rats (**Table 2** and **Supplemental Figure S12**). Furthermore, *Bifidobacteriaceae* and the associated phylotype PT33 (closely related to *B. longum*) were distinctly more abundant in distal fecal samples than in proximal fecal samples (**Supplemental Figure S12** and **Supplemental Table S3**), strengthening the assumption of specific shifts in gut microbiota composition during intestinal transit independent of the different doses of administered E2. *Peptostreptococcaceae, Turicibacteraceae*, and *Clostridiaceae* were significantly higher in Sham-Vehicle rats when compared to OVX-Vehicle rats (**Table 2**) reinforcing aforementioned findings (**Figure 1**), which indicated that the relative abundance of phylotypes in these two families was lower in rats that lacked ovarian hormones after OVX, including E2 (**Figure 1**).

**Table 2:** Families that displayed significantly (LEfSe) higher relative 16S rRNA gene abundances in Sham-Vehicle rats compared to OVX-Vehicle, OVX-E2-Low, or OVX-E2-High rats^a^.

	OVX-Vehicle	OVX-E2-Low	OVX-E2-High
**Fecal**			
** Proximal**	*Bifidobacteriaceae* *Peptostreptococcaceae* *Turicibacteraceae*	*-*	*-*
** Distal**	*Bifidobacteriaceae* *Peptostreptococcaceae* *Turicibacteraceae* *Clostridiaceae*	*Bifidobacteriaceae* *Erysipelotrichaceae*	*Bifidobacteriaceae* *Erysipelotrichaceae*
**Mucosal**			
** Proximal**	*Turicibacteraceae*	–	–
** Distal**	*Turicibacteraceae*	–	*Bifidobacteriaceae*

a Group abbreviation (e.g., Sham-Vehicle) are described in **Table 1**.

### Impact of Surgical Menopause With or Without E2 Treatment on Spatial Memory, Obesity-Related Measures, and Peripheral Markers of Estrogen Stimulation

**Figure 4:**
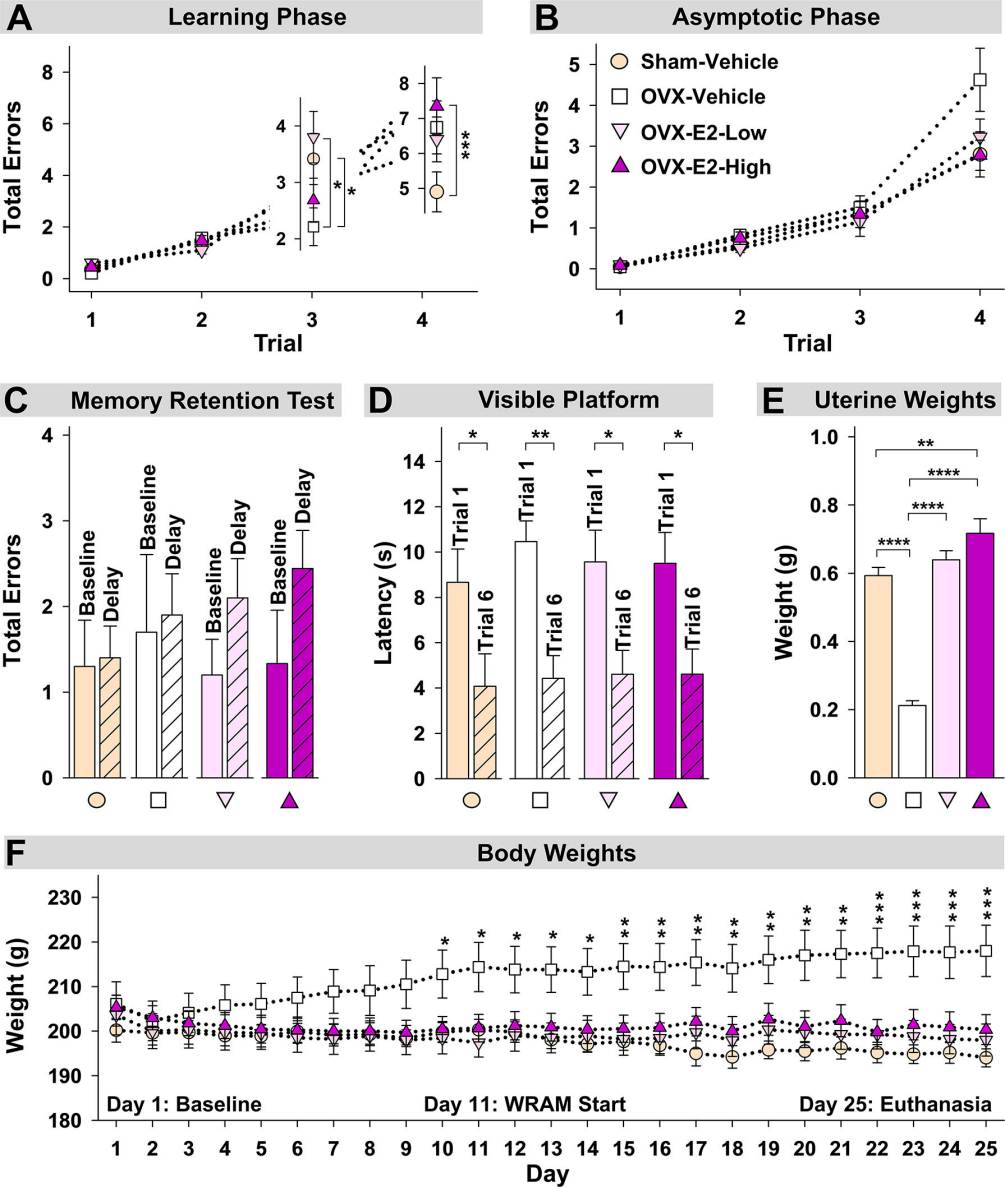
Spatial memory and obesity markers. **(A)** During the Learning Phase, Trial x Treatment interactions suggested that as trials increased, patterns of group performance changed. On Trial 3, the OVX-Vehicle group showed enhanced performance compared to the Sham group and compared to the OVX-E2-Low group, while on Trial 4, the ovary-intact Sham group outperformed the Ovx-E2-High group and tended to outperform the OVX-Vehicle and OVX- E2-Low groups, indicating an OVX-induced impairment at the maximum working memory load that E2 treatment did not attenuate. **(B)** By the end of WRAM testing, all subjects performed similarly across trials. **(C)** Each treatment group was not impaired following a four-hour delayed memory retention trial. **(D)** Each treatment group decreased latency to the visible platform from the first to last trial, indicating swimming, motoric, and visual competency. **(E)** OVX-Vehicle rats had decreased uterine weights compared to all other groups, indicating that surgery-induced estrogen loss results in a lack of uterine stimulation; E2 treatment after OVX successfully stimulates uterine tissue to that of ovary-intact rats. **(F)** Body weight increased in OVX-Vehicle rats beginning 10 days after surgery and remained elevated until the end of the experiment compared to all other groups. Group abbreviations (e.g., Sham-Vehicle) are described in **Table 1**. Error bars indicate SEM. The asterisks indicate significant differences (*p ≤ 0.05; **p ≤ 0.01; ***p ≤ 0.001; ****p ≤ 0.0001.

During the Learning Phase of the WRAM, a Trial x Treatment interaction was present for Total Errors committed across all trials for Sham-Vehicle *vs* OVX-Vehicle [F_(3,54)_ = 5.36, p < 0.01] (**Figure 4A**). When only high working memory load trials, Trial 3 and Trial 4, were assessed, a Trial x Treatment interaction was present again for Sham-Vehicle *vs* OVX-Vehicle [F_(1,18)_ = 9.05, p < 0.01] (**Figure 4A**). To further explore this effect, Trial 3 and Trial 4 were assessed independently. On the moderate load trial, Trial 3, OVX enhanced performance compared to ovary-intact Sham treatment [OVX-Vehicle *vs* Sham-Vehicle: F_(1,18)_ = 4.81, p < 0.05], while on the maximum load trial, Trial 4, OVX tended to impair performance compared to ovary-intact Sham treatment [OVX-Vehicle *vs* Sham-Vehicle: F_(1,18)_ = 3.40, p = 0.06], although this effect did not reach statistical significance for this comparison. During the Asymptotic Phase of the WRAM, there was a non-significant trend of an OVX-induced impairment on this high working memory load trial (**Figure 4B**). To determine if OVX indeed had an impairing effect compared to the ovary-intact Sham treatment that was pervasive throughout testing, we performed an additional analysis assessing Trial 4 only across Days 2-12, thereby collapsing across both phases. There was a significant effect of Treatment, where OVX impaired memory on this maximum working memory load trial compared to Sham treatment [F_(1,18)_ = 7.23, p < 0.05].

Regarding estrogen effects, during the Learning Phase of the WRAM, a Trial x Treatment interaction was present for Total Errors committed across all trials for Sham-Vehicle *vs* OVX-E2-High [F_(3,51)_ = 10.01, p < 0.0001], and OVX-E2-Low *vs* OVX-E2-High [F_(3,51)_ = 3.19, p < 0.05] comparisons, and a marginal effect for OVX-Vehicle *vs* OVX-E2-Low [F_(3,54)_=2.60, p = 0.06] (**Figure 4A**). When only high working memory load trials, Trial 3 and Trial 4, were assessed, a Trial x Treatment interaction was present again for Sham-Vehicle *vs* OVX-E2-High [F_(1,17)_ = 17.05, p < 0.001], and OVX-E2-Low *vs* OVX-E2-High [F_(1,17)_ = 6.06, p < 0.05] comparisons (**Figure 4A**), and a marginal effect for OVX-Vehicle *vs* OVX-E2-Low [F_(3,54)_=3.32, p = 0.09]. For Trial 3, OVX alone made fewer errors compared to the OVX-E2-Low group [F_(1,18)_ = 6.60, p < 0.05]. For Trial 4, the OVX-E2-High group made more errors compared to the Sham-Vehicle group [F_(1,17)_ = 15.57, p < 0.001], and the OVX-E2-Low group tended to make more errors compared to the Sham-Vehicle group [F_(1,18)_ = 3.48, p =0.08].

By the Asymptotic Phase, all groups successfully learned to solve the WRAM task in a similar fashion regardless of estrogen treatment. The four-hour delayed memory retention test on Day 13 of the WRAM task revealed that none of the treatment groups had significantly impaired working memory on Trial 3 following a four-hour delay compared to their baseline scores on the last regular testing session (**Figure 4C**).

Following completion of the WRAM task, rats were assessed on the Visible Platform, a control task that confirms swimming, motoric, and visual capacity. For each treatment group, latency to platform (seconds) on the first *vs* last trial was compared. A main effect of Trial was present for each group (Sham-Vehicle: [F_(9,1)_ = 5.55, p < 0.05], OVX-Vehicle: [F_(9,1)_ = 19.76, p < 0.01], OVX-E2-Low: [F_(9,1)_ = 8.28, p < 0.05], OVX-E2-High: [F_(8,1)_ = 9.34, p < 0.05]; **Figure 4D**). Across all groups, the average latency to the visible platform on Trial 1 was 9.55 ± 0.634 seconds *vs* an average latency to the visible platform of 4.43 ± 0.561 seconds on Trial 6. Overall, these data indicate that all subjects, regardless of treatment group, had sufficient motor and visual capacity to perform the procedural components of water-escape maze tasks.

Regarding vaginal cytology, a peripheral indicator of estrogen stimulation, ovary-intact (Sham-Vehicle) rats exhibited normal estrous cyclicity or evidence of slightly elongated estrous cycles across the three-day evaluation period, which is a common phenomenon in middle-aged intact rats around 12 months of age (for review, see: Wise et al., 1997; Kermath and Gore, 2012; Koebele and Bimonte-Nelson, 2016). OVX-Vehicle rats had diestrus-like or blank smears, indicating successful removal of the ovaries. OVX-E2-Low and OVX-E2-High rats displayed estrus-like smears with cornified cells across all evaluation days, indicating successful stimulation of the vaginal epithelium resulting from daily E2 treatment following OVX.

Uterine weights obtained after euthanasia, another peripheral marker of estrogen stimulation, indicated a significant effect of Treatment [F_(3,35)_ = 64.76, p < 0.0001] (**Figure 4E**). Fisher’s PLSD *post hoc* test indicated that OVX rats without E2 treatment (i.e., the OVX-Vehicle group) had significantly lower uterine weights compared to ovary-intact Sham-Vehicle rats (p < 0.0001), OVX-E2-Low rats (p < 0.0001) and OVX-E2-High rats (p < 0.0001). Whereas ovary-intact Sham-Vehicle rats and OVX-E2-Low rats did not differ in uterine weight, OVX-E2-High rats had greater uterine weights compared to ovary-intact Sham-Vehicle rats (p < 0.01); moreover, OVX-E2-High rats had marginally greater uterine weights compared to the OVX-E2-Low group (p = 0.06), collectively indicating that 4 µg of daily E2 treatment after OVX likely leads to a slightly supra-physiological level of circulating E2 than would be expected in an ovary-intact rat or with a lower dose of E2 treatment after OVX.

**Figure 5:**
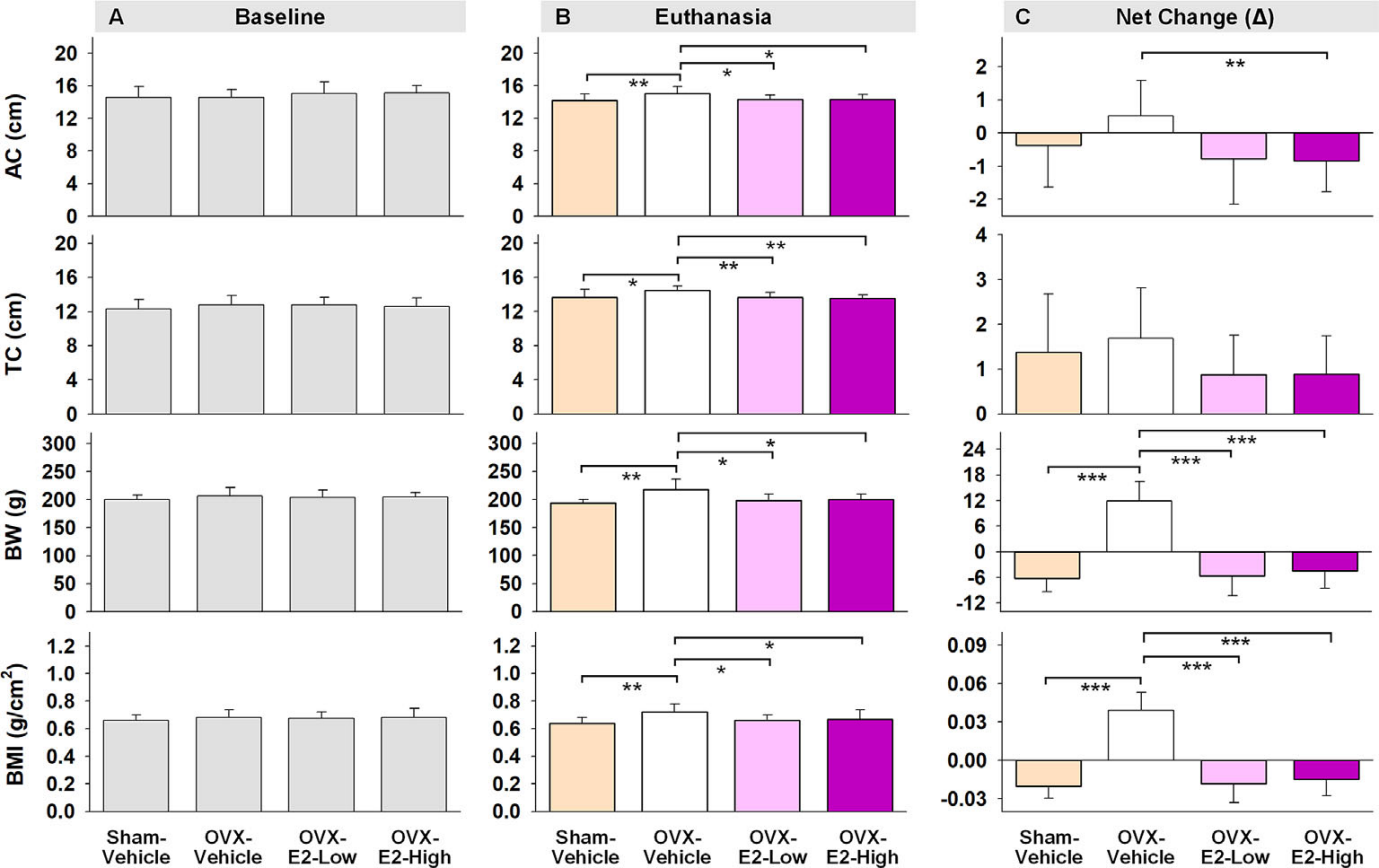
Abdominal circumferences (AC), thoracic circumferences (TC), body weight (BW), and BMI at the beginning of experiment (**A**, Baseline), at the end of experiment (**B**, Euthanasia), and net changes **(C)**. The asterisks indicate significant differences (*p ≤ 0.05; **p ≤ 0.01; ***p ≤ 0.001; pairwise comparison using Fisher’s PLSD *post hoc* test after ANOVA). Group abbreviations (e.g., Sham-Vehicle) are described in **Table 1**. Error bars indicate SEM.

Regarding obesity markers, at the beginning of the study, prior to surgery and hormone treatments, there were no differences in body length, abdominal circumference, thoracic circumference, body weight, or BMI among rats (**Figure 5A**). At euthanasia, there was a significant effect of Treatment for circumference measures (Abdominal Circumference: [F_(3,35)_ = 3.25, p < 0.05]; Thoracic Circumference [F_(3,35)_ = 4.75, p < 0.01]) (**Figure 5B**). OVX-Vehicle rats had a significantly greater Abdominal Circumference compared to ovary-intact Sham-Vehicle rats (p < 0.01), OVX-E2-Low rats (p < 0.05) and OVX-E2-High rats (p < 0.05). OVX-Vehicle rats also had a greater Thoracic Circumference compared to ovary-intact Sham-Vehicle rats (p < 0.01), OVX-E2-Low rats (p < 0.01) and OVX-E2-High rats (p < 0.01). Net change analyses indicated a marginal main effect of Treatment [F_(3,35)_ = 2.75, p = 0.057] for Abdominal Circumference, and no significant net change in Thoracic Circumference (**Figure 5C**). A repeated measures ANOVA analysis of Body Weight across days revealed a main effect of Treatment [F_(3,35)_ = 3.40, p < 0.05], a main effect of Day [F_(24,840)_ = 5.92, p < 0.0001], and a Treatment x Day interaction [F_(72,840)_ = 18.71, p < 0.0001]. The OVX-Vehicle group weighed significantly more than ovary-intact Sham-Vehicle rats (p < 0.01), OVX-E2-Low rats (p < 0.05), and OVX-E2-High rats (p < 0.05) in the overall analysis (**Figure 4F**). When body weight was analyzed separately *via* ANOVA for each evaluation day, a significant effect of Treatment became apparent 10 days after surgery [F_(3,35)_ = 3.25, p < 0.05], and continued this pattern through to euthanasia [F_(3,35)_ = 7.37, p < 0.001], where the OVX-Vehicle group weighed significantly more than any other group (**Figure 4F**). At euthanasia, there was also a significant effect of Treatment on Body Weight [F_(3,35)_ = 7.37, p < 0.001] and BMI [F_(3,35)_ = 4.23, p < 0.01] (**Figure 5B**). Fisher’s PLSD *post hoc* indicated that Body Weight (g) was increased in OVX-Vehicle rats compared to Sham-Vehicle rats (p < 0.0001), OVX-E2-Low rats (p < 0.001), and OVX-E2-High rats (p < 0.01). Likewise, Fisher’s PLSD *post hoc* for BMI showed that OVX-Vehicle rats had an increased BMI compared to Sham-Vehicle rats (p < 0.01), OVX-E2-Low rats (p < 0.05), and OVX-E2-High rats (p < 0.05). Net weight change analyses also revealed a main effect of Treatment for Body Weight [F_(3,35)_ = 44.30, p < 0.0001] and BMI [F_(3,35)_ = 48.83, p < 0.0001].

### Exploratory Correlations Among Gut Microbiota, BMI, and Cognitive Scores

**Figure 6:**
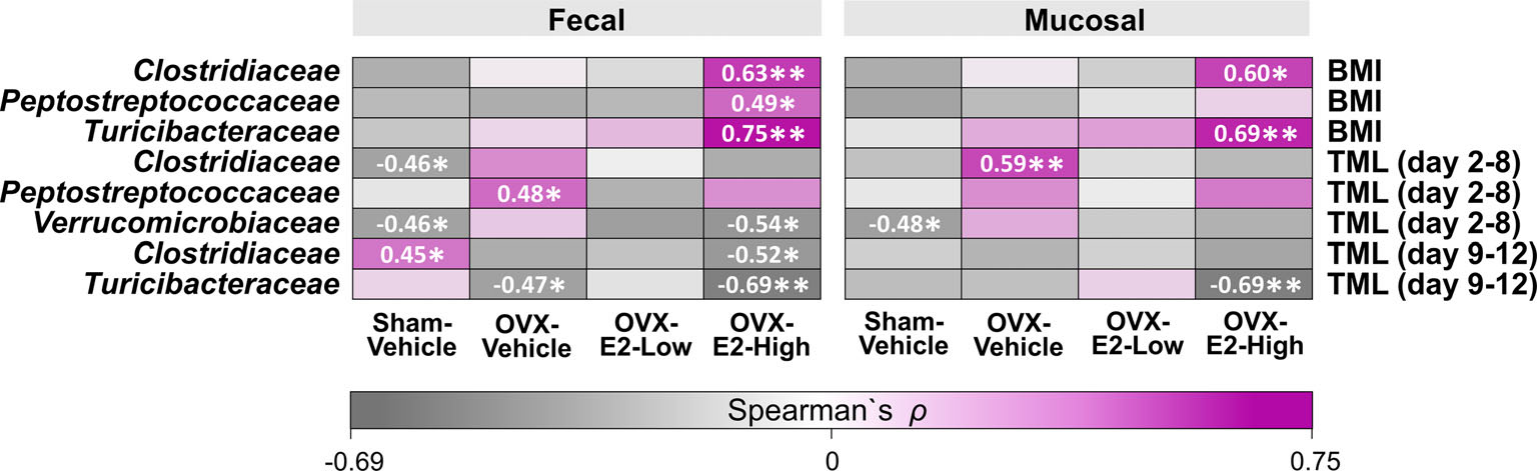
Heatmap of positive and negative correlations for each Treatment group between intestinal microbial families and Body mass index (BMI) at the end of experiment as well as cognitive data (Total Maximum Load [TML]) during Learning and Asymptotic Phases of the WRAM. The asterisks indicate significant differences (*p ≤ 0.05; **p ≤ 0.01) whereas the shades and numbers indicate the degree of correlation. Group abbreviations (e.g., Sham-Vehicle) are described in **Table 1**.

Based on studies that illustrated potential interactions between gut microbiota and body weight as well as cognitive function (John et al., 2018; Mohajeri et al., 2018; Sun et al., 2020), we evaluated exploratory Spearman ρ correlations for each treatment group individually between intestinal microbial families that were significantly affected by OVX and E2 treatments (**Figure 1**) and either (a) BMI based on body weight at the end of the experiment or (b) error scores at the highest working memory load trial, trial 4 (Total Maximum Load [TML] trial) during the Learning Phase as well as the Asymptotic Phase of WRAM testing (**Figure 6**). In fecal samples, significant positive correlations were revealed for BMI within the OVX-E2-High treated group, where a greater BMI was associated with a higher relative abundance of *Clostridiaceae* (*p* < 0.01; ρ = 0.63), *Peptostreptococcaceae* (*p* < 0.05; ρ = 0.49), and *Turicibacteraceae* (*p* < 0.01; ρ = 0.75). In mucosal samples, BMI positively correlated with higher relative abundance of *Clostridiaceae* (*p* < 0.05; ρ = 0.60) and *Turicibacteraceae* (*p* < 0.01; ρ = 0.69), such that a greater BMI was associated with higher relative abundance of these microbial families specifically in the OVX-E2-High group (**Figure 6**). BMI did not correlate with microbial families at a sufficient strength to reach statistical significance within the ovary-intact Sham-Vehicle group, OVX-Vehicle group, or OVX-E2-Low group.

Cognitive score correlations detected with the intestinal microbial gut families of interest were more variable than BMI correlations. During the Learning Phase of testing, wherein we observed a Trial x Treatment interaction driven by high working memory load trials, Spearman ρ analysis revealed a negative correlation within the Sham-Vehicle group between the number of errors made on the maximum working memory load trial and the relative abundance of *Clostridiaceae* (*p* < 0.05; ρ = -0.46) in the fecal samples and *Verrucomicrobiaceae* in both fecal (*p* < 0.05; ρ = -0.46) and mucosal (*p* < 0.05; ρ = -0.48) samples, such that better memory performance was associated with a higher relative abundance of *Clostridiaceae* and *Verrucomicrobiaceae* (**Figure 6**). Better memory performance was also associated with a higher relative abundance of *Verrucomicrobiaceae* (*p* < 0.05; ρ = -0.54) in the fecal samples of the OVX-E2-High group during the Learning Phase. The OVX-Vehicle group had a positive correlation between maximum working memory load trial error scores during the Learning Phase and relative abundance of *Peptostreptococcaceae* in the fecal samples (*p* < 0.05; ρ = 0.48) and *Clostridiaceae* in the mucosal samples (*p* < 0.01; ρ = 0.59), such that poorer memory performance was related to a higher relative abundance of *Peptostreptococcaceae* and *Clostridiaceae* (**Figure 6**).

During the Asymptotic Phase of the WRAM, when rats were performing to the best of their ability, several correlations between gut composition and memory scores were revealed. For the ovary-intact Sham-Vehicle group, there was a positive correlation between maximum memory load error scores and *Clostridiaceae* in the fecal samples (*p* < 0.05; ρ = 0.45), where poorer memory was associated with a higher relative abundance of *Clostridiaceae.* For the OVX-Vehicle group, there was a negative correlation between maximum memory load error scores and *Turicibacteraceae* in fecal samples, such that OVX-Vehicle rats that made more errors had a lower relative abundance of *Turicibacteraceae* present in fecal samples (*p* < 0.05; ρ = -0.47). In the OVX-E2-High group, there was also a negative correlation between maximum memory load error scores during the Asymptotic Phase and relative abundance of *Clostridiaceae* (*p* < 0.05; ρ = -0.52) in the fecal samples, and with *Turicibacteraceae* in both fecal (*p* < 0.01; ρ = -0.69) and mucosal samples (*p* < 0.01; ρ = -0.69), where poorer working memory was associated with a lower relative abundance of these microbial families (**Figure 6**). Additional correlations between BMI and memory scores with other microbial families *not* significantly affected by OVX and E2 treatments can be found in **Supplemental Figure S13**.

## Discussion

The relationship between an increased risk of health disorders and the loss of ovarian function is well established and thought to be mediated, in part, by menopause-associated declines in endogenous hormones such as E2 and progesterone (Rosano et al., 2007; Vegeto et al., 2008; Brand et al., 2013; Lizcano and Guzmán, 2014). Indeed, several studies have demonstrated relationships between E2 deficiency and Alzheimer’s and Parkinson’s diseases, breast and colon cancer, diabetes type 2, obesity, and cardiovascular diseases (Trichopoulos et al., 1972; Beaud et al., 2005; Rosano et al., 2007; Vegeto et al., 2008; Brand et al., 2013; Lizcano and Guzmán, 2014; Gagnière, 2016). It is important to note that although menopause typically occurs in parallel with aging, age alone does not account for the significantly increased risk of developing these negative health outcomes in post-menopausal women, indicating that the loss of ovarian hormones plays a unique role in the risk, development, and progression of cardiovascular, neurodegenerative, and other diseases (Parker et al., 2009; Faubion et al., 2015; Hale and Shufelt, 2015; Riedel et al., 2016; Benjamin et al., 2017; El Khoudary and Thurston, 2018; El Khoudary et al., 2018; El Khoudary et al., 2019; El Khoudary et al., 2020). These diseases are also each linked to diverse compositional changes of the intestinal microbiota involving common bacterial families related to the phyla *Firmicutes*, *Bacteroidetes*, and *Verrucomicrobia* (Koeth et al., 2013; Branchereau et al., 2016; Vogt et al., 2017; Ticinesi et al., 2018; Kazemian et al., 2020). Although compositional changes in the gut microbiota are known to be involved in the mediation of E2 levels (Kwa et al., 2016; Baker et al., 2017; Tetel et al., 2018) the potential intersectional links between menopause, E2 levels, gut microbiota, and negative health outcomes are less understood. The findings presented herein provide novel insight into the connections among ovarian hormone loss and replacement, gut microbiota regulation, and higher order cognitive function, effectively linking endocrine and gastrointestinal function to a multitude of health-related outcomes and opening new avenues of research in these previously distinct fields.

### E2-Linked Changes in Gut Microbiota and Potential Impact on Overall Health

The 16S rRNA gene sequence analysis indicated that the four common intestinal fermentative families *Clostridiaceae*, *Turicibacteraceae*, *Peptostreptococcaceae*, and *Porphyromonadaceae* were affected by OVX-induced E2 deficiency and subsequent E2 treatment. In this regard, the abundance of all four families displayed a gradual increase as the administered dose of E2 increased from low to high after OVX. In a clinical study, a decrease in *Clostridiaceae, Turicibacteraceae, Peptostreptococcaceae* was observed in Alzheimer’s disease patients compared to a neurotypical control group, suggesting that such compositional alterations might be linked to patterns of neurodegenerative diseases in humans (Vogt et al., 2017). A higher abundance of *Porphyromonadaceae* has been linked to cognitive decline, cardiovascular disease, and diabetic sensitivity (Branchereau et al., 2016; Ticinesi et al., 2018; Kazemian et al., 2020). However, members of *Clostridiaceae* and *Peptostreptococcaceae* are also positively associated with TMAO (trimethylamine N-oxide) blood levels in humans, an amine oxide involved in cardiovascular diseases (Koeth et al., 2013). These contradictory effects on health illustrate the challenge of finding the sensitive balance of administered E2, as well as highlight the complexity of taking individual health histories and risk factors into account when administering a treatment with the aim of producing optimal health outcomes. Furthermore, it is important to acknowledge that health outcomes might be personalized, species-specific, and/or species-dependent, and that further methodical exploration in this area of research is needed.

Here, the compositional changes of the intestinal gut microbiota were concomitant with changes in the SCFA profile. Specifically, the relative abundance of isobutyrate gradually increased with an increased dose of E2 after surgical menopause, whereas the OVX-induced increase in formate gradually decreased with an increased dose of E2 after surgical menopause. Furthermore, the relative abundance of these fermentation products in Sham-Vehicle rats were most similar to those detected in OVX-E2-Low rats, suggesting that the low E2 dose optimally compensated for the loss of ovaries. Isobutyrate is a branched chain fatty acid (BCFA) produced by gut fermenters when the branched amino acid valine is available (Zarling and Ruchim, 1987), and has been negatively associated with obesity (Ilhan et al., 2017). BCFAs can (a) affect the gastrointestinal motility and (b) accumulate in host serum where they potentially affect the central nervous system (e.g., as a neuromodulator) (Van Treuren and Dodd, 2020). BCFAs, and more specifically isobutyrate, was low in morbidly obese patients before gastric bypass surgery, and concentrations increased after surgery as patients successfully lost weight (Ilhan et al., 2020). Furthermore, BCFAs might affect (a) cholesterol synthesis, (b) mitochondrial β-oxidation of pyruvate, and (b) lipogenesis in adipocytes (Zabin and Bloch, 1951; Gregersen, 1979; Heimann et al., 2016; Van Treuren and Dodd, 2020), factors which are associated with an increased risk of obesity, metabolic syndrome, and diabetes (Van Treuren and Dodd, 2020).

Intestinal formate, produced by fermentative anaerobes in the gut, contributes 50% to the total circulating formate pool in mammals (Pietzke et al., 2020). Formate is generally used to fulfill the single-carbon molecules demand to synthesize nucleotides and methyl groups but alterations in formate metabolism potentially cause several disorders (Pietzke et al., 2020). Formate is also known to act as potential neurotoxin (Kapur et al., 2007) which additionally has inhibitory effects on the cytochrome C oxidase (last enzyme of the respiratory chain), supporting the assumption that organs that have a high oxygen demand such as the brain, heart, and kidney can be damaged by increased formate concentrations (Liesivuori and Savolainen, 1991). Overall, altered relative abundances of isobutyrate and formate following OVX may be a key influence in our observed obesity-related and cognitive outcomes and should be further investigated in future experiments.

The analysis of predicted microbial pathways revealed that the abundance of microbes associated with orthologs of the urea cycle and the polyamine biosynthesis (e.g., putrescine, spermidine, and spermine) increased when E2 was present, whether in ovary-intact Sham-Vehicle rats or in OVX rats that received E2 treatment after ovary removal. The administration of E2 after OVX has previously been shown to stimulate certain enzymes activities, including the carbamoylphosphate synthase, a urea cycle key enzyme (Lamers and Mooren, 1981). This E2-linked stimulation of the urea cycle might increase the formation of polyamides (e.g., putrescine, spermidine, and spermine) since ornithine and arginine, direct polyamine precursors, are intermediates of the urea cycle (LeMoine and Walsh, 2015). Polyamines have a broad range of biological health-affecting functions in the host. For example, they are involved in cell division and regenerative growth, neuroprotection, tumorigenesis, skin pathophysiology, lipid metabolism, fertility, and the integrity of pancreas, liver, heart, and central nervous system (Jänne et al., 2005; Pegg, 2009; Minois et al., 2011). Furthermore, they improve the integrity of the intestine, promoting intestinal restitution and increasing mucus secretion, and inhibit the production of proinflammatory cytokines (Oliphant and Allen-Vercoe, 2019). However, these compounds also show the potential to (a) cross the blood-brain barrier or (b) initiate its disruption (Shin et al., 1985; Glantz et al., 1996; Diler et al., 2002), which may increase the risk of neurodegeneration due to alterations in brain polyamine levels (Morrison and Kish, 1995) and an increased influx of neurotoxic debris, cells, and microbial pathogens into the brain (Sweeney et al., 2018), respectively.

The findings of the current study also indicated an E2-linked significant increased abundance of microbes in the fecal samples that potentially harbor enzymes for sucrose degradation. Indeed, phylotypes that were closely related to species that are known to ferment sucrose, such as *R. ilealis, C. celatum*, and *P. goldsteinii* (Hauschild and Holdeman, 1974; Sakamoto and Benno, 2006; Gerritsen et al., 2014), also showed an increased abundance concomitant to (a) the administration of E2 and (b) predicted sucrose degradation. As mentioned above, E2 can affect the activity of certain enzymes, including several host-produced intestinal enzymes such as sucrase (Hamden et al., 2011). Based on the assumption that administered E2 concentrations are higher in the intestinal host tissue than inside the gut lumen, the host-produced sucrase is potentially more affected by administered E2 than the sucrase produced by bacteria in the middle of the gut lumen. This theoretical possibility suggests that diet-driven sucrose, which is normally hydrolyzes by host-produced sucrase and subsequently absorbed, remains in the gut and serves as substrate for fermentative microbes, which most likely caused the observed increase of predicted sucrose degradation. Based on these considerations, a lack of estrogen resulting from surgical removal of the ovaries potentially improves the activity of host-produced sucrase and thus the absorption of associated monosaccharides, which in turn might contribute to the observed weight gain in OVX rats that did not receive exogenous E2 treatment.

Statistical analysis of differences between rats with intact ovaries and rats without ovaries that received E2 treatment indicated lower relative abundance of the common intestinal families *Bifidobacteriaceae* (dominated by a phylotype closely related to *Bifidobacterium longum*) and *Erysipelotrichaceae* (dominated by a phylotype closely related to *Clostridium cocleatum*) in rats administered low or high doses of E2 after OVX. Notably, several associations have been observed in humans with regard to health outcomes and *Bifidobacteriaceae*. For example, a decreased abundance of gut-barrier protecting *Bifidobacteriaceae* was observed in patients with neurodegenerative diseases (Vogt et al., 2017), whereas probiotics including *Bifidobacterium longum* improved anxiety and depression related behaviors in human participants (Messaoudi et al., 2011), suggesting potential neuroprotective properties of *Bifidobacteriaceae*. Enhanced abundance of these taxa is also associated with improvements in insulin sensitivity, lipid profile, blood pressure, weight loss, anti-inflammatory signals, stress levels, and short-term memory (O’Mahony et al., 2005; O’Hara et al., 2006; Xiao et al., 2013). The abundance of *Erysipelotrichaceae* was found to be decreased in patients with Alzheimer’s and cardiovascular diseases (Jie et al., 2017; Li et al., 2018). Furthermore, a higher abundance of *Clostridium cocleatum* might be involved in an improved intestinal anti-pathogen barrier (Wildeboer-veloo et al., 2003) collectively pointing to protective properties conferred by *Erysipelotrichaceae*.

### Cognitive Outcomes and Obesity-Related Measures

The loss of ovarian hormones *via* surgical menopause can detrimentally impact performance on some complex cognitive tasks (Daniel, 2013; Luine, 2014; Frick, 2015; Koebele and Bimonte-Nelson, 2015; Korol and Pisani, 2015). Whether hormone treatment, and particularly E2, improves cognition with surgical menopause depends on a multitude of factors, including age, dose, route of administration, duration of treatment, cognitive domains evaluated, and menopause etiology (Koebele and Bimonte-Nelson, 2015). Furthermore, there are few comprehensive cognitive evaluations in middle-aged female rats that also include an ovary-intact Sham control group as an additional reference point. Here, during the Learning Phase of the WRAM, Sham-Vehicle rats made more errors on the moderate working memory trial compared to OVX-Vehicle counterparts, an effect we have observed previously that may be associated with exploratory behavior during task learning (Koebele et al., 2019). Importantly, when working memory load was maximally taxed, ovary-intact Sham rats tended to make fewer errors compared to OVX-Vehicle counterparts, suggesting a surgically menopausal-induced cognitive impairment during learning. The Sham-Vehicle group also exhibited enhanced spatial memory performance when memory load was sufficiently burdened compared to the OVX-E2-High group, and marginally better performance compared to the OVX-E2-Low group, indicating that neither the daily low dose nor the high dose of E2 utilized in this experiment was sufficient to recapitulate the enhanced cognitive phenotype of age-matched ovary-intact rats.

While there was no statistical difference detected in memory performance between groups in the Asymptotic Phase alone, there was a trend toward an OVX-induced memory impairment. Thus, when we combined the learning and asymptotic phases, OVX-Vehicle-treated rats made significantly more errors compared to ovary-intact Sham rats across all testing days. These findings demonstrate that surgical menopause significantly impaired spatial learning and memory compared to age-matched ovary-intact Sham rats in a pervasive manner extending throughout testing, and that the detrimental effects of surgical menopause superseded potential benefits of E2 treatment during learning of a complex cognitive task. The delayed memory retention test did not indicate group impairments; a longer delay between trials may have been necessary to detect an OVX-induced impairment in memory retention, which has been previously been reported in our laboratory and others under other experimental parameters (Luine et al., 1998; Harburger et al., 2009; Witty et al., 2012; Prakapenka et al., 2018; Koebele et al., 2020b).

Prior research has suggested that there may be interactions between age and E2 dose for cognitive outcomes, such that E2 dose may need to be increased as age increases in order to produce similar effects compared to age-matched controls (Foster, 2003). Furthermore, there is evidence that substantial daily handling of rats can obscure the beneficial effects of E2 treatment in OVX rats assessed in an eight-arm radial maze task (Bohacek and Daniel, 2007); the relatively short duration of this experiment in conjunction with the required significant handling for injections, daily body weight measures, and cognitive testing may have contributed to the lack of E2 effect in surgically menopausal rats in the WRAM task. It is notable that the low and high doses of E2 did produce significant changes in gut microbiota composition following OVX; however, E2 treatment alone after OVX did not completely mimic the gut microbial community profile of ovary-intact rats. Thus, it would be prudent to investigate a broader range of E2 doses, combination hormone therapies, alternative routes of administration, and variation in experimental timeline parameters in the future to better understand the optimal hormone treatment regimen to not only beneficially impact gastrointestinal health, but also have a desirable effect on higher order cognitive processes.

Hormone treatment was initiated 48 hours after surgery for translational relevance, as women who undergo surgical menopause typically begin hormone therapy immediately to prevent unwanted symptoms associated with abrupt hormone loss. As a peripheral marker of hormone loss or E2 stimulation after OVX, vaginal cytology assessments were completed within one week of surgery. The three-day vaginal cytology monitoring period confirmed that OVX-Vehicle-treated rats had diestrus-like or blank smears, indicative of successful ovary removal. OVX-E2-Low and OVX-E2-High groups exhibited vaginal smears with cornified cells across all days, confirming systemic circulation of E2, including stimulation of the vaginal epithelium. Sham-Vehicle rats exhibited normal cyclic changes in vaginal smears across days, suggesting that ovarian function remained intact after Sham surgery and estrous cyclicity was evident. At the end of the study, uterine weights confirmed that Sham-Vehicle, OVX-E2-Low, and OVX-E2-High rats had heavier uteruses compared to OVX-Vehicle rats, which is another indicator of E2 activity.

Body weight is well-documented to increase following OVX and decrease with E2 treatment (Kakolewski et al., 1968; Bell and Zucker, 1971; Wade, 1972; Geary et al., 1994; Westerlind et al., 1998; Engler-Chiurazzi et al., 2012; Cox-York et al., 2015; Prakapenka et al., 2018; Koebele et al., 2020b), although gut microbial composition has not been systematically evaluated in conjunction with weight change after OVX and E2 treatment until now. At the beginning of the experiment, prior to surgery and initiation of hormone treatment, there were no statistical differences in abdominal circumference, thoracic circumference, body weight, or BMI among rats. Daily body weight assessment revealed a clear OVX-induced weight gain within 10 days of ovary removal that persisted through the end of the experiment, while low or high dose E2 administration to OVX rats prevented this weight gain. Indeed, at the end of the experiment, the OVX-Vehicle group had significantly increased abdominal and thoracic circumference measures, increased body weight, and increased BMI compared to the ovary-intact Sham-Vehicle group as well as both the OVX-E2-Low and OVX-E2-High groups, indicating a clear role for ovarian hormone loss and subsequent E2 treatment in weight regulation, visceral adipose distribution, and overall BMI. These observations corroborate findings in the clinical literature indicating increased relative risks of metabolic syndrome and obesity following surgical or transitional menopause, and also closely relate to the increased risk for the development of disorders such as diabetes type 2, cardiovascular disease and coronary heart disease in menopausal women (Farrag et al., 2002; Rocca et al., 2007; Rosano et al., 2007; Vegeto et al., 2008; Rocca et al., 2012; Brand et al., 2013; Lizcano and Guzmán, 2014; Neves-e-Castro et al., 2015; Baber et al., 2016; NAMS, 2017).

### Potential Relationships Between Gut Microbiota, BMI, and Cognition

Lastly, exploratory correlations among gut microbial families impacted by OVX and E2 treatment with BMI and cognitive outcomes provide novel insight into the gut-brain connection. In particular, fecal and mucosal samples from the OVX-E2-High group revealed several significant positive correlations between BMI and common gut microbes; specifically, a greater body weight at euthanasia within this group was associated higher relative abundance of *Clostridiaceae* and *Turcibacteraceae* in fecal and mucosal samples, as well as higher relative abundance of *Peptostreptococcaceae* in fecal samples only, highlighting that individual differences can influence relationships between gut microbiota and obesity outcomes within a treatment group. *Firmicutes* and the associated families *Clostridiaceae* and *Peptostreptococcaceae* have been reported to be more abundant in the gut of obese individuals than in normal-weight individuals (Ley et al., 2005; Nirmalkar et al., 2018). It is important to recall that the OVX-E2-High rats had overall higher abundance of *Clostridiaceae* (*Firmicutes)*, *Turicibacteraceae* (*Firmicutes*), and *Peptostreptococcaceae* (*Firmicutes*) compared to OVX-Vehicle rats, which may have promoted the presence of stronger correlations in general. Bacterial families within the *Firmicutes* phyla have a diverse range of activities in the gut. The ratio of *Firmicutes* to *Bacteroidetes* phyla have been reported to be increased with reproductive senescence and stroke in a rat model (Park et al., 2020). A higher *Firmicutes* to *Bacteroidetes* ratio has also been associated with obesity outcomes as a result of changes in metabolic processes and serum hormone levels in humans (Turnbaugh et al., 2006; Clarke et al., 2012; Shin et al., 2019). As such, although the OVX-E2-High group had similar overall body weight to Sham-Vehicle and OVX-E2-Low groups, relationships among BMI and gut microbial composition suggest that individual within-group differences could influence overall health and multiple divergent mechanisms may be at play depending on physiological status that eventually produce similar phenotypes. Correlations with BMI were not present with sufficient strength to reach statistical significance within the ovary-intact Sham-Vehicle group, OVX-Vehicle group, or OVX-E2-Low group. This suggests that a higher E2 dose in a surgically menopausal background results in unique relationships between gut microbiota and BMI. This underscores the complexity of endocrine-gastrointestinal system interactions and, translationally, highlights the need for a personalized approach to healthcare matters that take into consideration individual life histories and personal factors that will influence eventual health outcomes.

Correlations between memory scores and relative abundance of microbial gut families are less clear. For example, within ovary-intact Sham rats, a higher relative abundance of *Clostridiaceae* and *Verrucomicrobiaceae* were associated with better memory during the Learning Phase of the WRAM, but *Clostridiaceae* was associated with worse memory during the Asymptotic Phase of the WRAM. For rats without their ovaries and no subsequent hormone treatment (i.e., the OVX-Vehicle group), a higher relative abundance of *Peptostreptococcaceae* in the fecal samples and *Clostridiaceae* in the mucosal samples were associated with poorer memory during the Learning Phase of the WRAM, and a lower relative abundance of *Turicibacteraceae* was associated with poorer memory during the Asymptotic Phase of the WRAM. These OVX-Vehicle rats showed higher body mass and strong positive correlation of *Peptostreptococcaceae* with error scores when working memory was maximally taxed*;* as these bacteria have been found abundant in obese population (Nirmalkar et al., 2018), this might explain why obesity is associated with worse memory performance through gut microbiota (Alarcón et al., 2016). Rats treated with the low E2 dose did not exhibit significant relationships between microbial families of interest and cognitive performance. However, rats treated with the high E2 dose showed better memory scores associated with higher abundance of fecal *Verrucomicrobiaceae* during the Learning Phase, as well as better memory scores related to higher abundance of fecal *Clostridiaceae* and *Turicibacteraceae* in both fecal and mucosal samples in the Asymptotic Phase. As such, E2 treatment after OVX results in unique relationships between gut composition and cognitive performance that differ from those observed in ovary-intact rats. It is important to note, however, that there are more specific mediating factors that moderate the observed relationships, and that the family-level analysis may be too broad for interpretation at this point. Future investigations should include a more detailed and reliable species-specific evaluation by, for example, using metagenome analysis. However, this first step in exploration had opened the door to new paths of discovery to uncover the intricacies of endocrine-gastrointestinal-central nervous system interactions. In this regard, in the future, additional assessments could address the mediating impact of OVX surgery itself *versus* effects of E2 treatment following surgical menopause to elucidate potential interactions between phylotypes and cognitive and obesity outcomes.

### Conclusions, Limitations, and Future Perspectives

**Figure 7:**
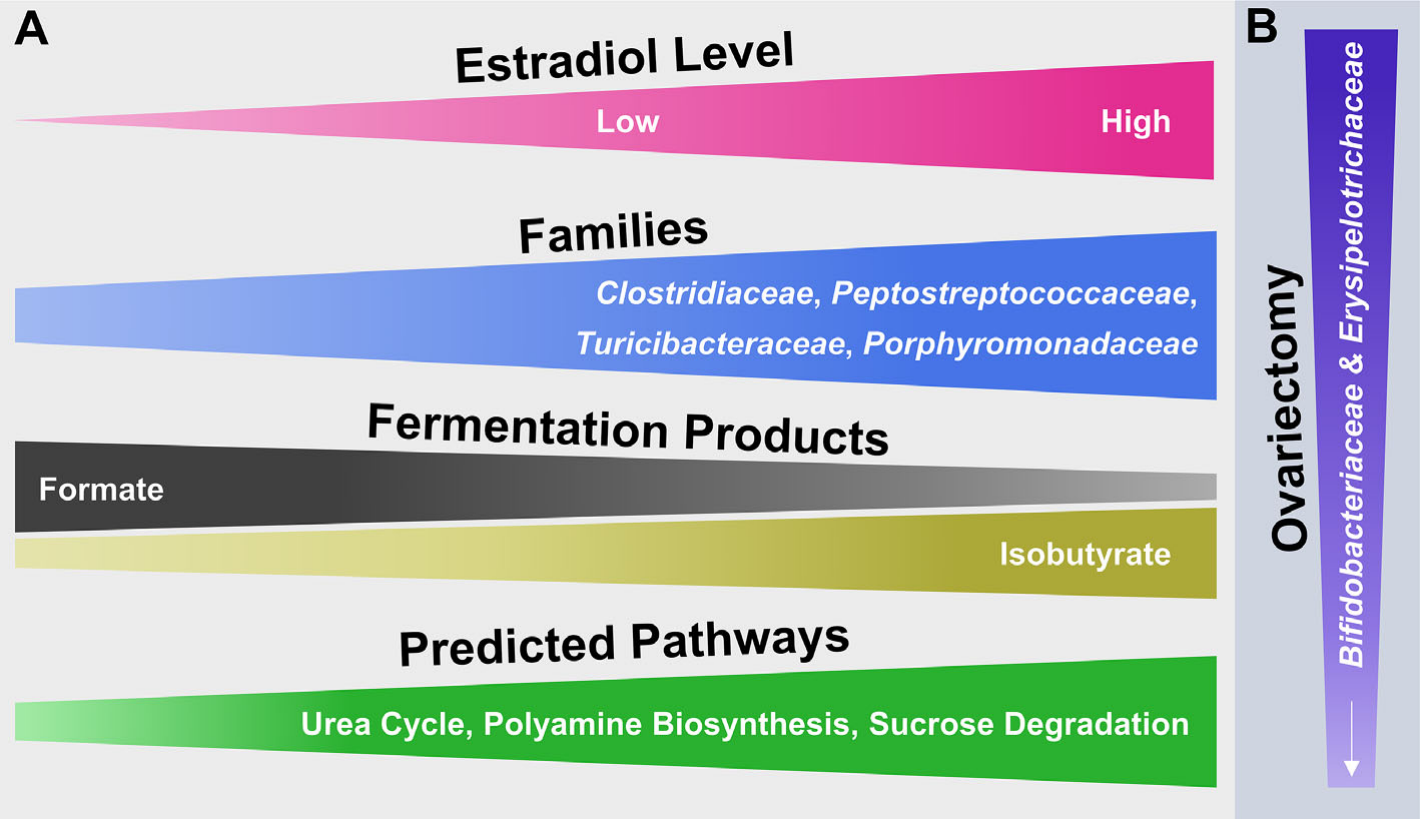
Hypothetical model illustrating the potential impact of E2 including endogenous levels as well as different exogenous doses following ovariectomy on microbial families of the mammalian gut microbiota and their functionality. **(A)** From left to right, increasing or decreasing width illustrates an increase or decrease in abundance of the respective attribute (e.g., fermentation products). **(B)** From top to bottom, decreasing width illustrates a decrease in abundance of microbial families.

The collective findings of this study examine surgical menopause-induced physiological and behavioral changes in relation to E2-linked compositional changes in the intestinal microbiota. The model that summarizes the main findings (**Figure 7**) is a theoretical abstraction and does not display all potential effects of surgical menopause and E2 hormone therapy administration on host health [e.g., E2 has anti-inflammatory properties independent of intestinal changes [Monteiro et al., 2014)]. Rather, the model emphasizes potential E2-linked alterations in the abundance of specific intestinal microbial taxa and their overall function, which most likely contribute to the enhanced risk of diseases or comorbidities associated with menopause and other forms of ovarian hormone loss.

The relatively short read length of Illumina-obtained forward reads (240 bp) compromises an accurate species-level taxonomic classification in this study (Yarza et al., 2014; Singer et al., 2016). Therefore, the theoretical model (**Figure 7**) is limited to microbial family-level identities of the main taxa that responded to OVX or E2 treatment. Additionally, the primer-based detection of a phylotype sequence is conditional on the detection of a given target sequence. Furthermore, for simplicity the taxonomic analysis was restricted to the most abundant (≥ 2% relative 16S rRNA gene abundance) families and phylotypes. In this regard, it is possible that less abundant taxa might be of relevance and likewise contributed to the observed findings.

Although PICRUSt prediction can show high similarities to shotgun metagenome sequencing (Langille et al., 2013), this bioinformatic analysis is based on 16S rRNA gene sequence abundances and therefore is subject to the same limitations of detection and classification as mentioned above. However, the findings qualitatively illustrate the impact of OVX and subsequent E2 treatment on a specific subset of fermentative intestinal families and their activities which collectively might contribute to menopause-linked diseases.

Intestinal variabilities in microbial composition or SCFA profiles observed from subjects within a specific group (Sham-Vehicle, OVX-Vehicle, OVX-E2-Low, OVX-E2-High) could have been caused by recently re-ingested feces, which also affect the bile acid composition and xenobiotic conjugates such as *beta*-glucuronidase (Bogatyrev et al., 2020). Potential ways to avoid alterations in gut microbial composition from coprophagia would include using fecal collection tail cups to prevent access to boli (Bogatyrev et al., 2020), although this would be challenging in the context of a long-term behavioral study. To reduce cross contamination from cage mates, rats could be placed in solitary housing conditions; however, single housing of female rats results in a myriad of detrimental health effects on behavioral outcomes, including increased anxiety-like and depressive-like behavioral profiles and decreased exploratory behavior and cognitive performance. Thus, in an experimental design that incorporates behavioral outcomes, pair-housing is preferable (Pinelli et al., 2017). Intestinal-produced microbial *beta*-glucuronidase and *beta*-glucosidase are part of the estrobolome, and affect the circulation of E2 in the body by reactivating the compound (Ervin et al., 2019; Ma et al., 2020). In addition, enhanced *beta*-glucuronidase levels in blood plasma and serum have been associated with the occurrence of breast cancer (Whitaker, 1960; Minton et al., 1986), reinforcing the presence of a relationship between functional changes of the intestinal microbiota, breast cancer, and menopause (Trichopoulos et al., 1972; Beaud et al., 2005; Gagnière, 2016). In the current experiment, neither surgical menopause nor E2 treatment significantly affected the abundance of microbes harboring genes for these enzymes, which may be related to the surgical menopause etiology used herein and/or the route of estrogen administration. Systematic experimentation evaluating these putative impactful factors on this relationship provide exciting future directions for potential translational discovery.

The loss of ovary function affects circulating levels of not only E2, but additional ovary-derived hormones such as other estrogens, progesterone, and androgens (Koebele and Bimonte-Nelson, 2016). The findings in the current study demonstrate that rats with intact ovaries have a unique profile of beneficial gut microbiota, and this profile is altered following surgical menopause; furthermore, E2 administration alone after surgical menopause is not sufficient to compensate for the altered microbial gut profiles induced by complex interactions between ovarian-derived hormones and the intestinal microbiota. This assumption is reinforced by studies that show different health impacts when females received (a) only-estrogen or (b) estrogen and progestin (Grodstein et al., 1996; Schairer et al., 2000; Johnson et al., 2009). Given the marked potential of E2-driven alterations of the intestinal microbiota composition, other sex steroid hormones such as progestins alone or estrogen/progestin combinations might have a similar potential to alter the gut community composition. In this regard, the potential impact of other ovarian-derived hormones and hormone combinations occurring endogenously or *via* exogenous administration (in the context of hormonal contraceptives or menopausal hormone therapies, for example) on the mammalian gut microbiota has, to our knowledge, not been investigated. It is also important to consider the impact of dietary composition on outcomes. While the standard rat chow used in this experiment included isoflavone content, which can act as a phytoestrogen, the amount present was unlikely to impact overall gut composition or behavior over and above the significant effects of OVX and chronic E2 treatment; furthermore, the OVX-Vehicle treated rats exhibited blank vaginal smears, indicating a lack of estrogenic stimulation. Another potential factor influencing gut composition after OVX is the inflammatory response induced by surgery itself. A recent experiment found that in male Sprague-Dawley rats, several peripheral inflammatory markers remained elevated up to a week after surgical intervention (Cata et al., 2021). However, post-surgical inflammatory responses are considered to be relatively acute compared to the robust and persistent effect of OVX and chronic E2 treatment. Indeed, the NSAID administered at the time of surgery and the potent anti-inflammatory effects of E2 likely aid in post-surgical recovery and a return to baseline. Furthermore, the Sham surgery group serves as a control for the impact of surgery itself beyond the systematic manipulations implemented in the experiment. Future studies focusing on how varied hormone parameters, dietary composition, inflammatory responses, and menopause etiology impact the intestinal microbiota and overall health measures in females are important to further understand these complex relationships and potentially have a translational impact.

A challenge to systematically assessing prospective mechanisms of estrogen-gut-brain interactions in human subjects is the considerable amount of inherent heterogeneity when assessing the various life factors that contribute to gut health, including genetic, epigenetic, and environmental variables (Turnbaugh et al., 2006; Wos-Oxley et al., 2012). As such, rodents serve as valuable models in that they allow methodical investigation into biological underpinnings of phenomena that are traditionally difficult to assess in humans in a systematic fashion (Turnbaugh et al., 2006; Wos-Oxley et al., 2012; Nagpal et al., 2018). The ability for researchers to control for environmental factors in conjunction with the relative homogeneity in genetic variance in inbred strains allows for a clearer understanding of the direct effects of the treatment in question as well as its impact on physiology and cognitive changes (Nguyen et al., 2015; Laukens et al., 2016). Although many investigations into gut microbiota thus far have utilized germ-free or antibiotic-treated specific-pathogen free rodents (Wos-Oxley et al., 2012; Laukens et al., 2016), we opted to use wildtype rats as a translational element to our experimental design, embracing microbial diversity within and between subjects that inevitably works together at a systems level to influence overall outcomes. Of course, it is important to recognize that there are limitations to rodent models, and that some of the observations we have described here may not be replicated in full in humans as a result of species-dependent gastrointestinal anatomy, evolutionary adaptations within the gut-brain axis, and gut microbial populations that exist at differing relative abundances in rats and humans, though it has been recently suggested that some aspects of the rat gut microbiota may be more representative of human gut composition than mice (Wos-Oxley et al., 2012; Fritz et al., 2013; Nguyen et al., 2015; Flemer et al., 2017; Nagpal et al., 2018; Pan et al., 2018).

Acknowledging these limitations, the research herein has nonetheless yielded exciting new insights into connections among the gut, endocrine system, and brain, and a novel understanding of the role of surgical menopause and estrogen treatment on the brain, peripheral body, and behavior. In the future, it will be important to assess variations in menopause etiology (e.g., transitional *vs* surgical), as well as investigate different estrogen doses, routes of administration, and timing of drug treatment, as well as translationally relevant combinations of estrogen and progestogens in both surgical and transitional menopause models to determine the effects of other ovarian hormones and combined hormone treatments on gut microbiota, body weight modulation, and cognitive outcomes. Direct evaluation of circulating ovarian hormone levels may also provide insight to a golden-ratio of ovarian hormones in the context of optimal gut and brain health. Continued investigation into the intricate connections among menopause, ovarian hormone status, aging, and gut-health will undoubtedly reveal crucial insights into the development of many age- and menopause- associated diseases. In turn, new discoveries and treatments related to endocrine and gut health will lead to potentially postponing or preventing a variety of diseases that disproportionately affect aging women, including cardiovascular diseases, metabolic syndrome, neurodegenerative diseases, and beyond, with the ultimate aim to improve quality of life and healthy life expectancy as a whole for women throughout the lifespan.

## Data Availability Statement

The datasets presented in this study can be found in online repositories. The names of the repository/repositories and accession number(s) can be found below: https://www.ebi.ac.uk/ena, LR898068, LR898243. Cognitive and obesity marker data will be made available by the authors upon reasonable request. Requests to access those datasets should be directed to HB-N, bimonte.nelson@asu.edu.

## Ethics Statement

The animal study was reviewed and approved by Arizona State University Institutional Animal Care and Use Committee.

## Author Contributions

LZ: Conceptualization, data curation, formal analysis, methodology, project administration, software, supervision, validation, visualization, writing – original draft, writing-review and editing. SK: Conceptualization, data curation, formal analysis, investigation, methodology, project administration, software, supervision, validation, visualization, writing – original draft, writing – review and editing. VB: Data curation, investigation, validation, writing-review and editing. ZI: Conceptualization, data curation, investigation, methodology, project administration, supervision, validation, writing-review and editing. BD: Data curation, investigation, formal analysis, software, validation, writing-review and editing. SN-S: Investigation, validation, writing-review and editing. RN: Investigation, validation, writing-review and editing. JM: Data curation, investigation, supervision, validation, writing-review and editing. KN: Data curation, formal analysis, software, supervision, validation, writing-review and editing. JF: Conceptualization, funding acquisition, supervision, validation, writing-review and editing. AM: Conceptualization, funding acquisition, supervision, validation, writing-review and editing. RK-B: Conceptualization, funding acquisition, methodology, project administration, resources, supervision, validation, visualization, writing – original draft, writing-review and editing. HB-N: Conceptualization, funding acquisition, methodology, project administration, resources, supervision, validation, visualization, writing – original draft, writing-review and editing. All authors contributed to the article and approved the submitted version.

## Funding

HB-N: NIA (AG028084), state of Arizona, Arizona Department of Health Services (ADHS14-052688), NIH Alzheimer’s Disease Core Center (P30AG019610), Arizona State University Office of Knowledge Enterprise Development, College of Liberal Arts and Sciences, and Department of Psychology. JF, AM, RK-B, and HB-N of the Mayo Clinic/Arizona State University Consortium for Menopause, Obesity, and the Microbiome (MAC-MOM) were jointly awarded funding from the Ken and Linda Morris Weight and Wellness Solutions Program (KLMWWSP) to fund this project. RKB is funded by NIDDK (R01DK105829).

## Conflict of Interest

The authors declare that the research was conducted in the absence of any commercial or financial relationships that could be construed as a potential conflict of interest.

## Publisher’s Note

All claims expressed in this article are solely those of the authors and do not necessarily represent those of their affiliated organizations, or those of the publisher, the editors and the reviewers. Any product that may be evaluated in this article, or claim that may be made by its manufacturer, is not guaranteed or endorsed by the publisher.
