# Supplementary material for: Surgical Menopause and Estrogen Therapy Modulate the Gut Microbiota, Obesity Markers, and Spatial Memory in Rats
Source: Front Cell Infect Microbiol. 2021 Sep 30;11:702628. doi: 10.3389/fcimb.2021.702628 (PMC8515187; doi:10.3389/fcimb.2021.702628)
Supplement: Supplementary file 1 [file DataSheet_1.pdf]

Supplemental Figures:

Figure S1. Experimental design and timeline.

Figure S2. Fecal and mucosal tissue samples were extracted from the distal and proximal colon at euthanasia.

Figure S3. Rarefaction analyses of bacterial 16S rRNA gene sequences obtained from fecal and mucosal samples of differently treated rats.

Figure S4. Alpha diversity assessed with Faith Diversity index of the intestinal microbial community based on (A) Sham-Vehicle, OVX-Vehicle, OVX-E2-Low, and OVX-E2-High rats or (B) fecal, mucosal, distal and proximal samples.

Figure S5. Weighted UniFrac analysis (PCoA) of gut microbial community composition of (A) different intestinal sample sites and (B) of rats treated with different E2 doses.

Figure S6. Unweighted UniFrac analysis (PCoA) of gut microbial community composition of (A) different intestinal sample sites and (B) of rats treated with different E2 doses.

Figure S7. Relative abundances of the most abundant bacterial phyla in fecal and mucosal samples obtained from differently treated rats.

Figure S8. Intestinal phylotypes and relatives at the (A) species-level or genus-level (B) that displayed a significant difference (higher or lower relative abundances) in OVX-E2-High rats than in OVX-Vehicle rats.

Figure S9. Relative abundance of *Verrucomicrobiaceae* and phylotype PT 1 (closely related to *Akkermansia muciniphila*).

Figure S10. Predicted relative abundance of Beta-Glucuronidase and Beta-Glucosidase.

Figure S11. Predicted relative abundance of (A) two polyamide biosynthesis pathways and (B) two sucrose degradation enzymes.

Figure S12. Relative 16S rRNA gene abundance of *Bifidobacteriaceae* and *Erysipelotrichaceae* and associated phylotypes closely related to *B. longum* and *C. cocleatum.*

Figure S13. Heatmap of positive and negative correlations for each treatment group between cognitive data and BMI at the end of experiment, and microbial families that were not significantly affected by different E2 doses.

Supplemental Tables:

Table S1. Total amount of short chain fatty acids (SCFAs) detected in the proximal or distal fecal samples of each rat.

Table S2. Total and relative abundance of the short chain fatty acids detected in each group in fecal samples.

Table S3. Relative 16S rRNA abundance of *Bifidobacteriaceae* and *B. longum* (PT33) in fecal and mucosal samples, each obtained from distal and proximal gut locations.

Table S4. Families that had a ≥ 2% relative 16S rRNA gene abundances in at least one of the samples.

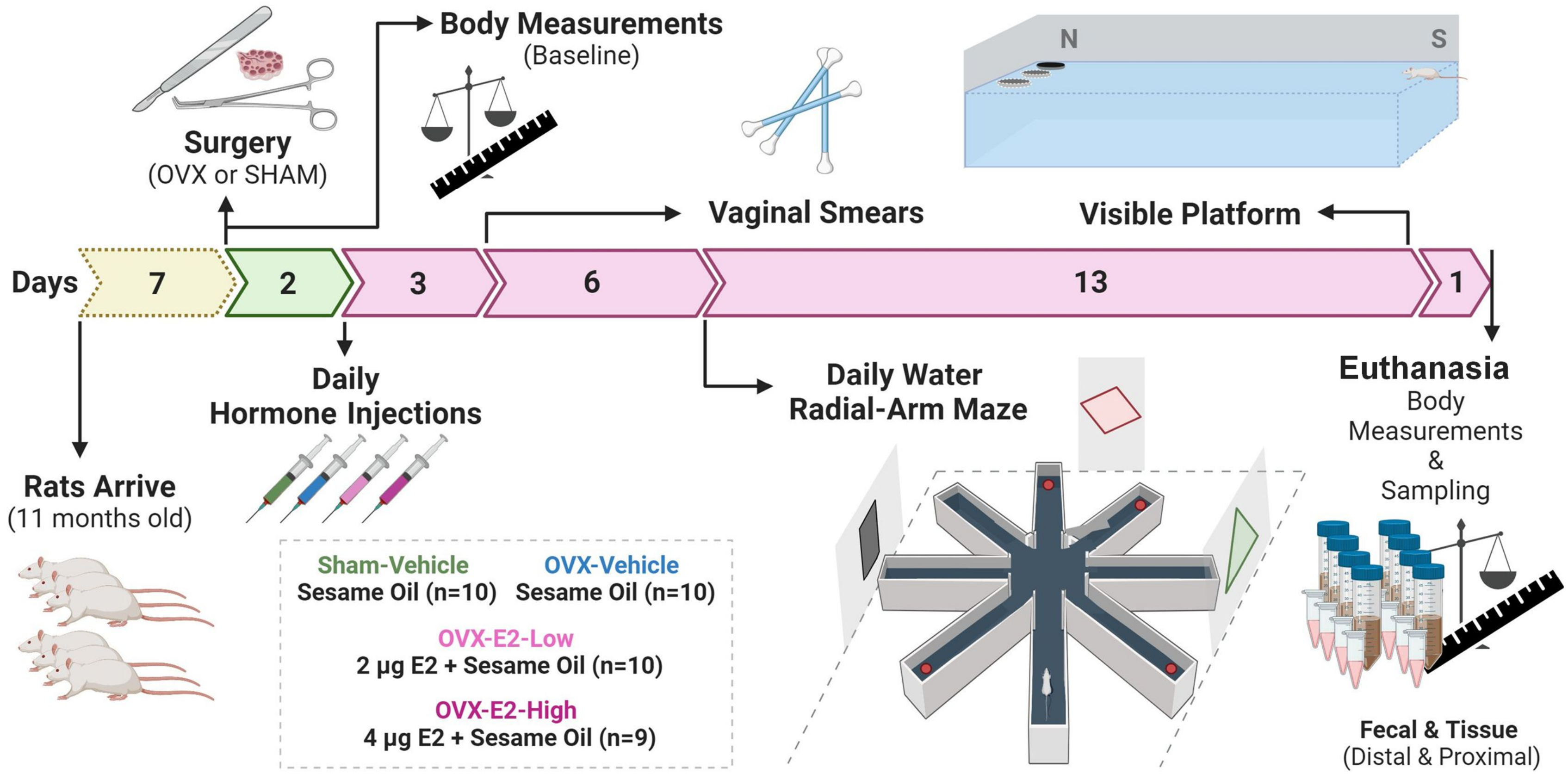

Figure S1. Experimental design and timeline. OVX or Sham surgery occurred one week after rat arrival. Daily Vehicle or E2 injections began 48 hours after surgery. Behavioral assays including the Water Radial-Arm Maze and Visible Platform task began 10 days after injections. Upon euthanization, obesity measures were obtained, and fecal and mucosal tissue samples were collected from distal and proximal colon for analysis.

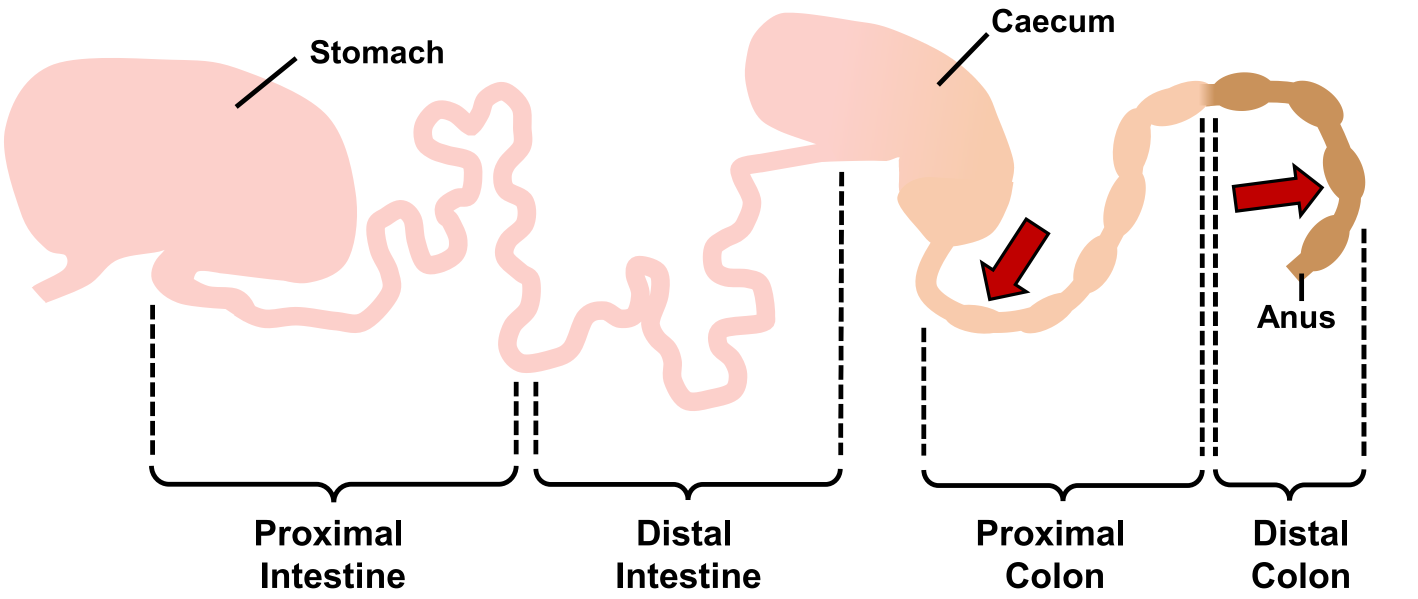

Figure S2. Fecal and mucosal tissue samples were extracted from the distal and proximal colon at euthanasia. Red arrows indicate the approximate location of the sampling site.

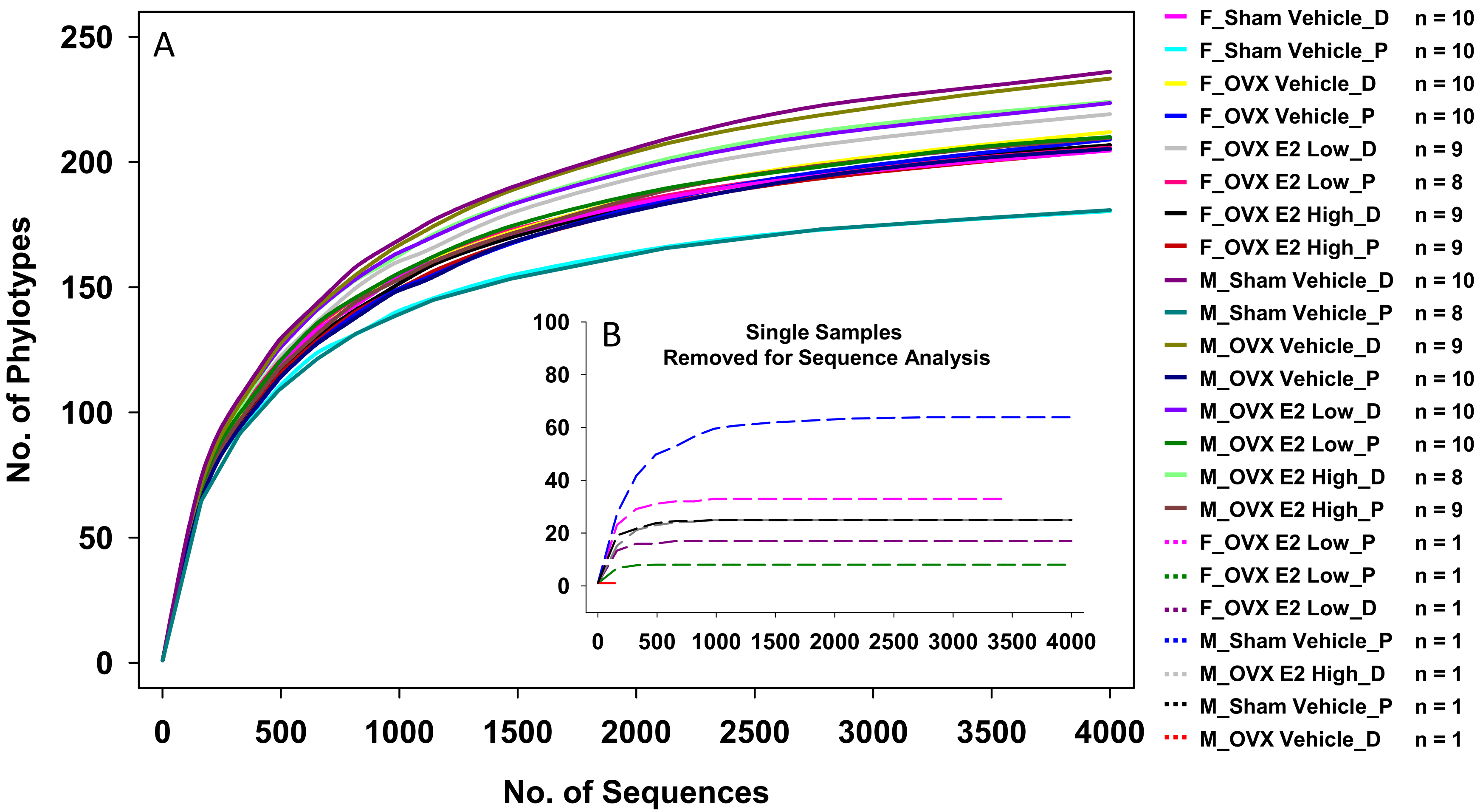

Figure S3. Rarefaction analyses of bacterial 16S rRNA gene sequences obtained from fecal and mucosal samples of differently treated rats. A, each curve represents the average of at least 8 samples. B, each curve represents a single sample. Samples shown in B were not considered. NOTE: one rat of the OVX E2 High group died following OVX surgery. Abbreviations: F, fecal; M, mucosal; D, distal colon sample; P, proximal colon sample; n, number of samples (replicates) used for each rarefaction curve. Group abbreviations (e.g., Sham-Vehicle) are described in Table 1.

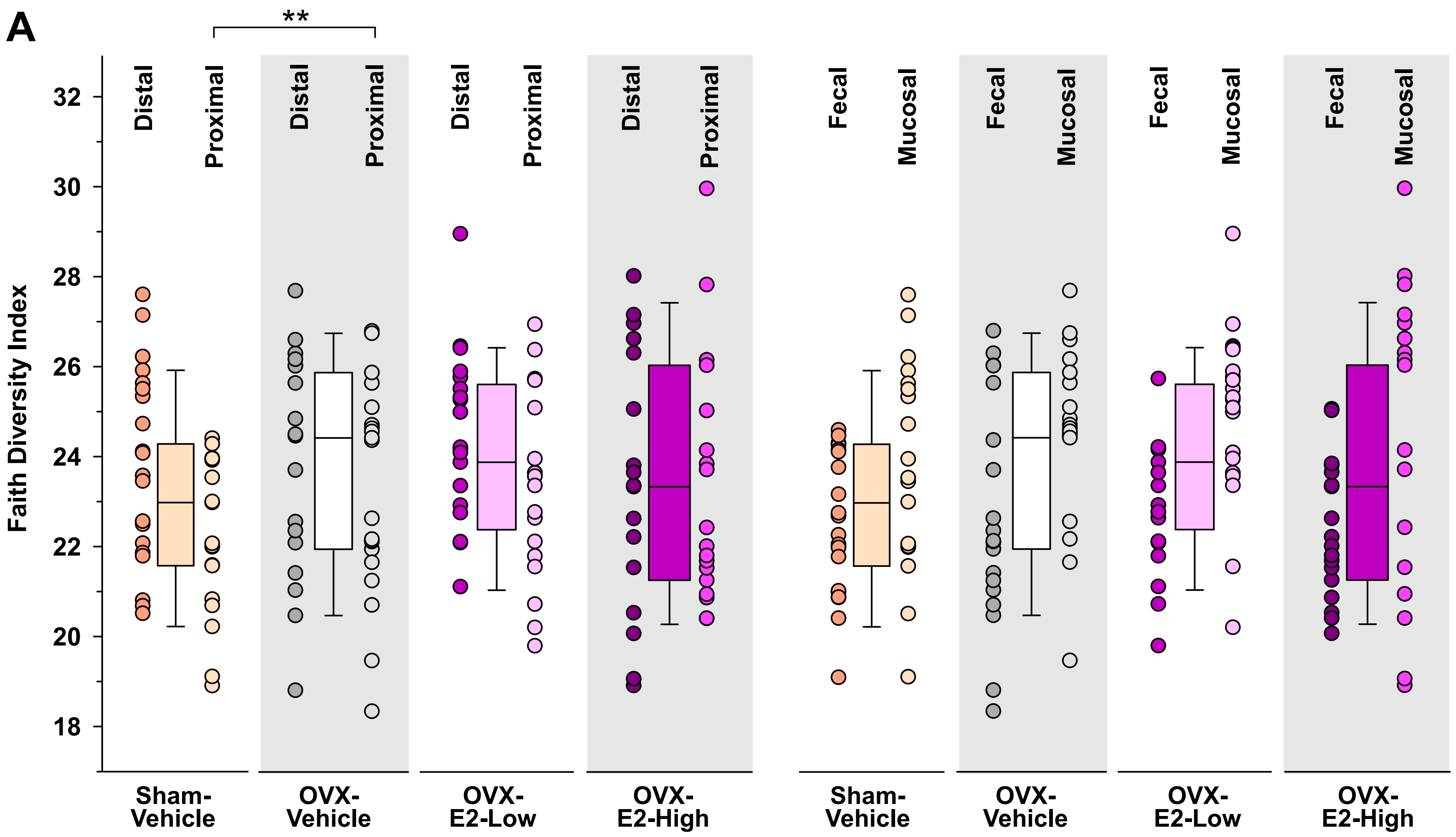

A
**
Faith Diversity Index
32
30
28
26
24
22
20
18
Distal
Proximal
Distal
Proximal
Distal
Proximal
Distal
Proximal
Fecal
Mucosal
Fecal
Mucosal
Fecal
Mucosal
Fecal
Mucosal
Sham-Vehicle
OVX-Vehicle
OVX-E2-Low
OVX-E2-High
Sham-Vehicle
OVX-Vehicle
OVX-E2-Low
OVX-E2-High

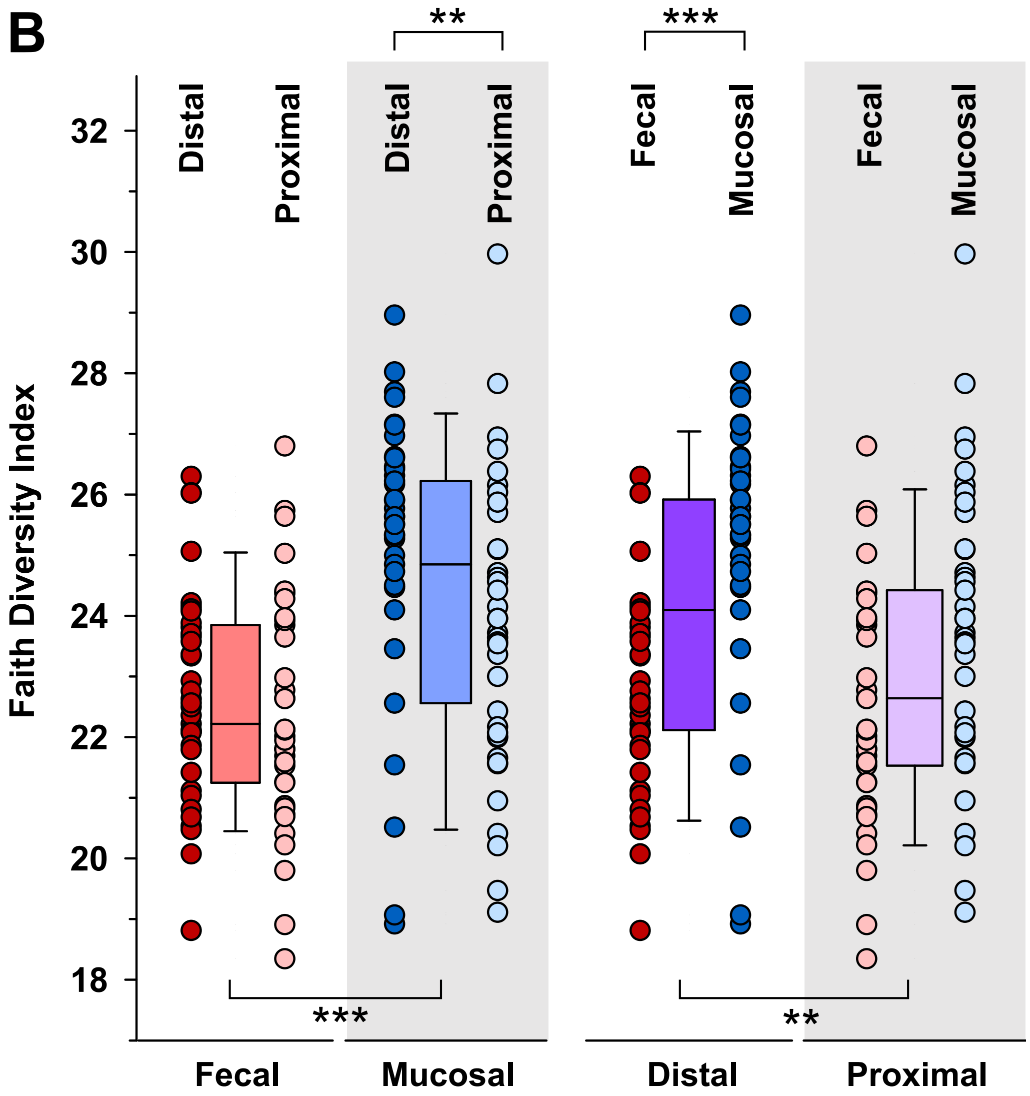

Figure S4. Alpha diversity assessed with Faith Diversity index of the intestinal microbial community based on (A) Sham-Vehicle, OVX-Vehicle, OVX-E2-Low, and OVX-E2-High or (B) fecal, mucosal, distal and proximal samples. The asterisks indicate significant differences between the Faith's phylogenetic diversity indices (** $p \leq 0.01$, *** $p \leq 0.001$, non-parametric pairwise comparison using Wilcoxon). Error bars indicate standard deviations.

.

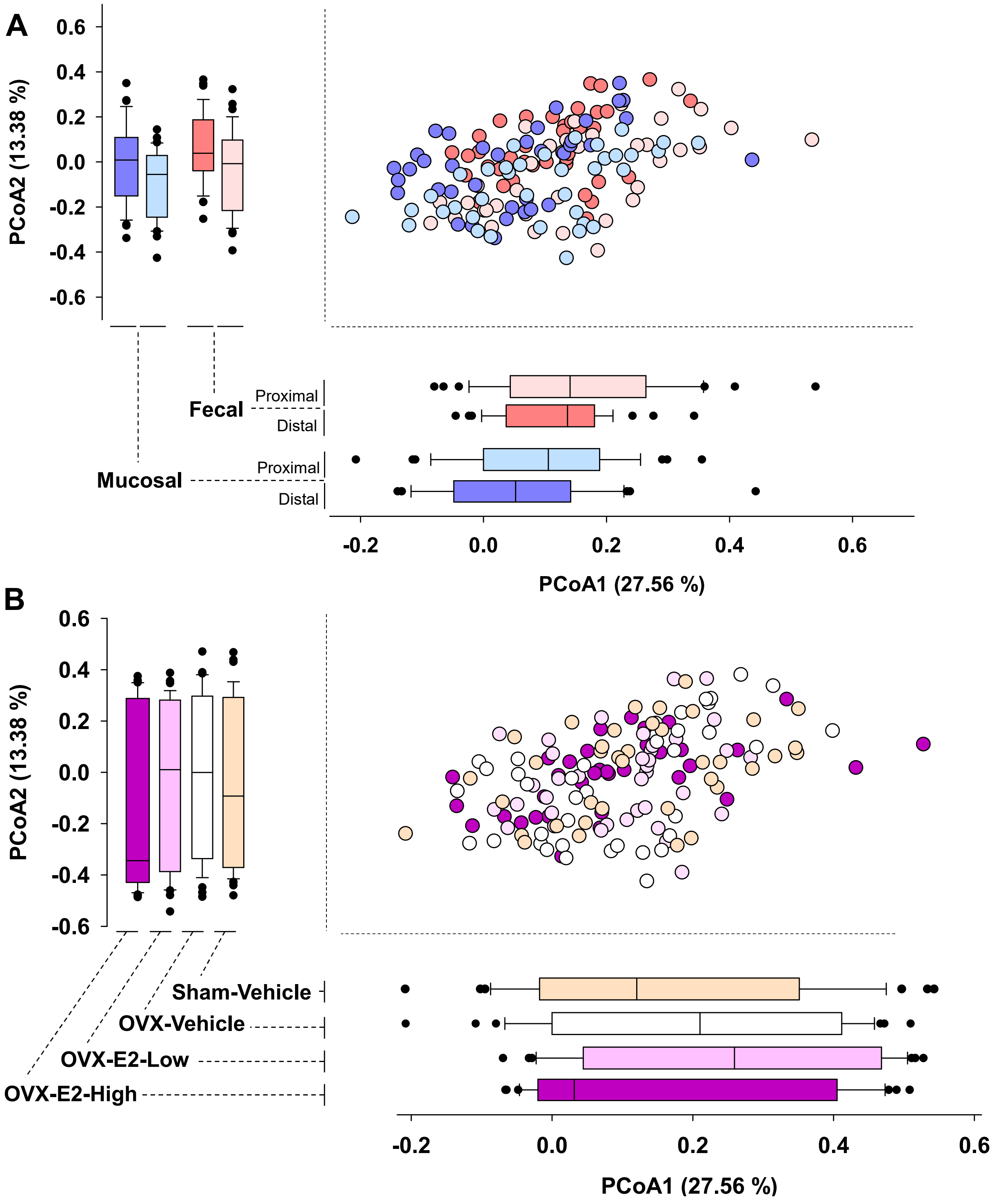

Figure S5. Weighted UniFrac analysis (PCoA) of gut microbial community composition of (A) different intestinal sample sites and (B) of rats treated with different E2 doses. Analysis based on 16S rRNA gene sequence. Group abbreviations (e.g., Sham-Vehicle) are described in Table 1. Error bars indicate standard deviations.

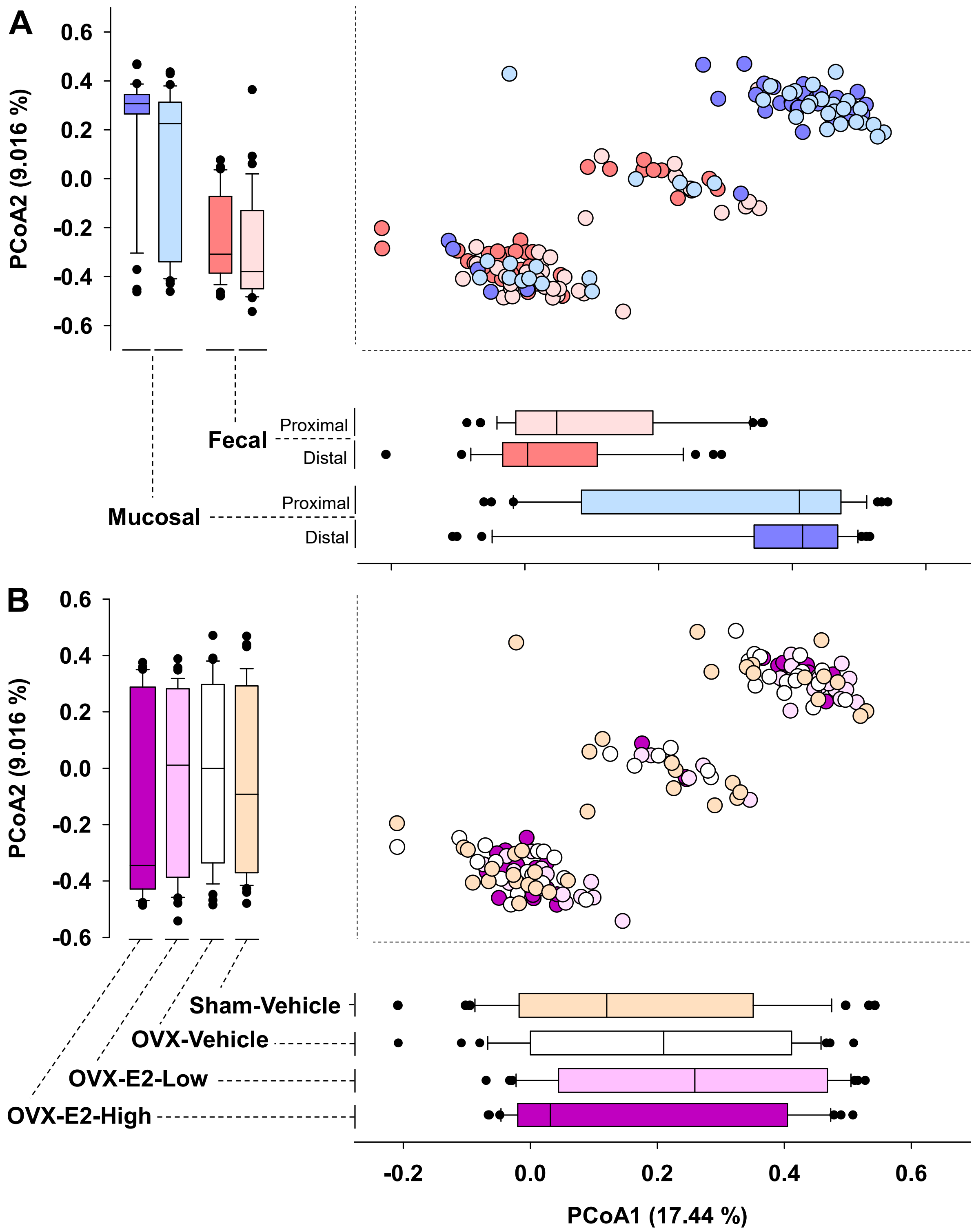

Figure S6. Unweighted UniFrac analysis (PCoA) of gut microbial community composition of (A) different intestinal sample sites and (B) of rats treated with different E2 doses. Analysis based on 16S rRNA gene sequence. Group abbreviations (e.g., Sham-Vehicle) are described in Table 1. Error bars indicate standard deviations.

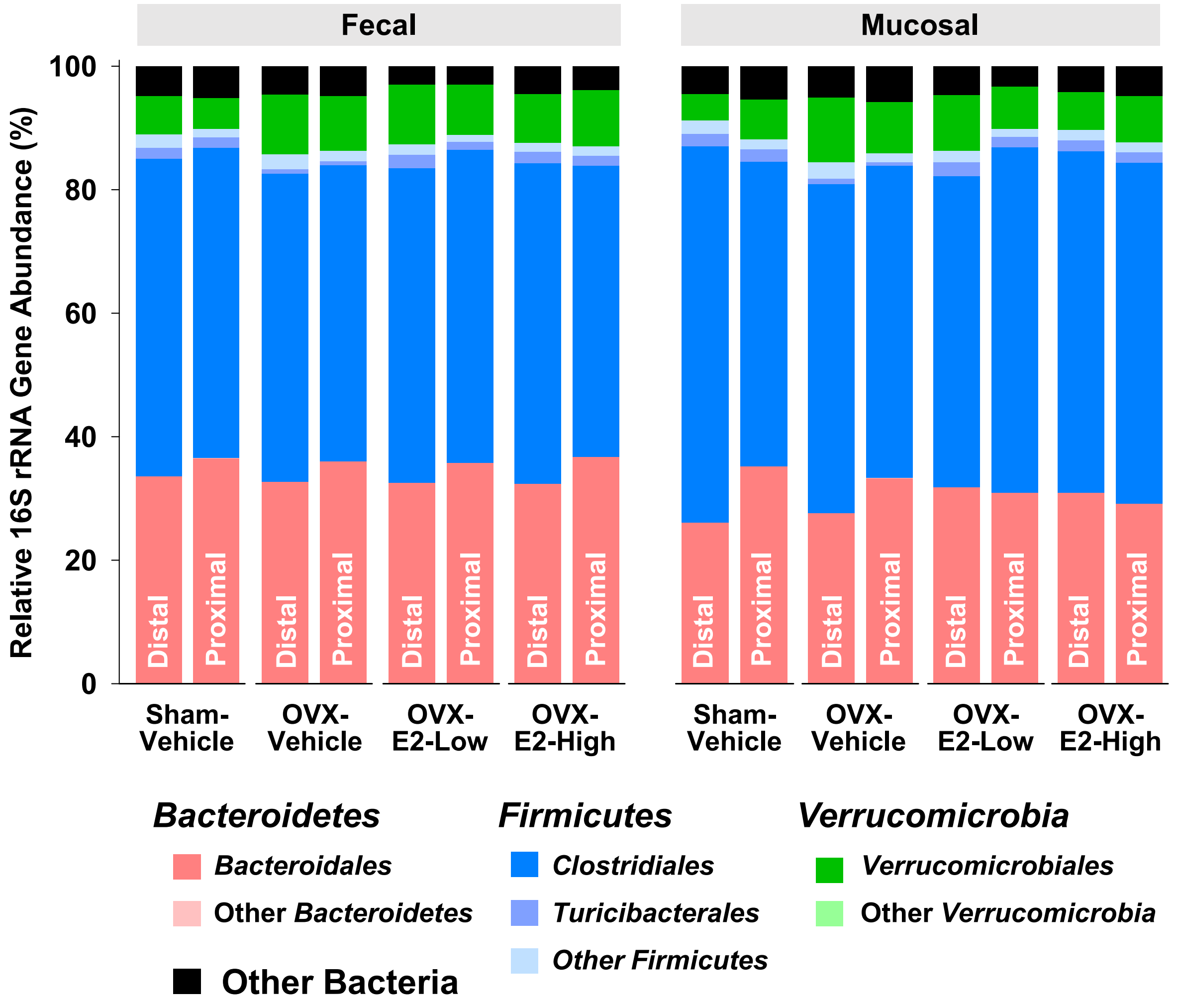

Figure S7. Relative abundances of the most abundant bacterial phyla in fecal and mucosal samples obtained from differently treated rats. Group abbreviations (e.g., Sham-Vehicle) are described in Table 1.

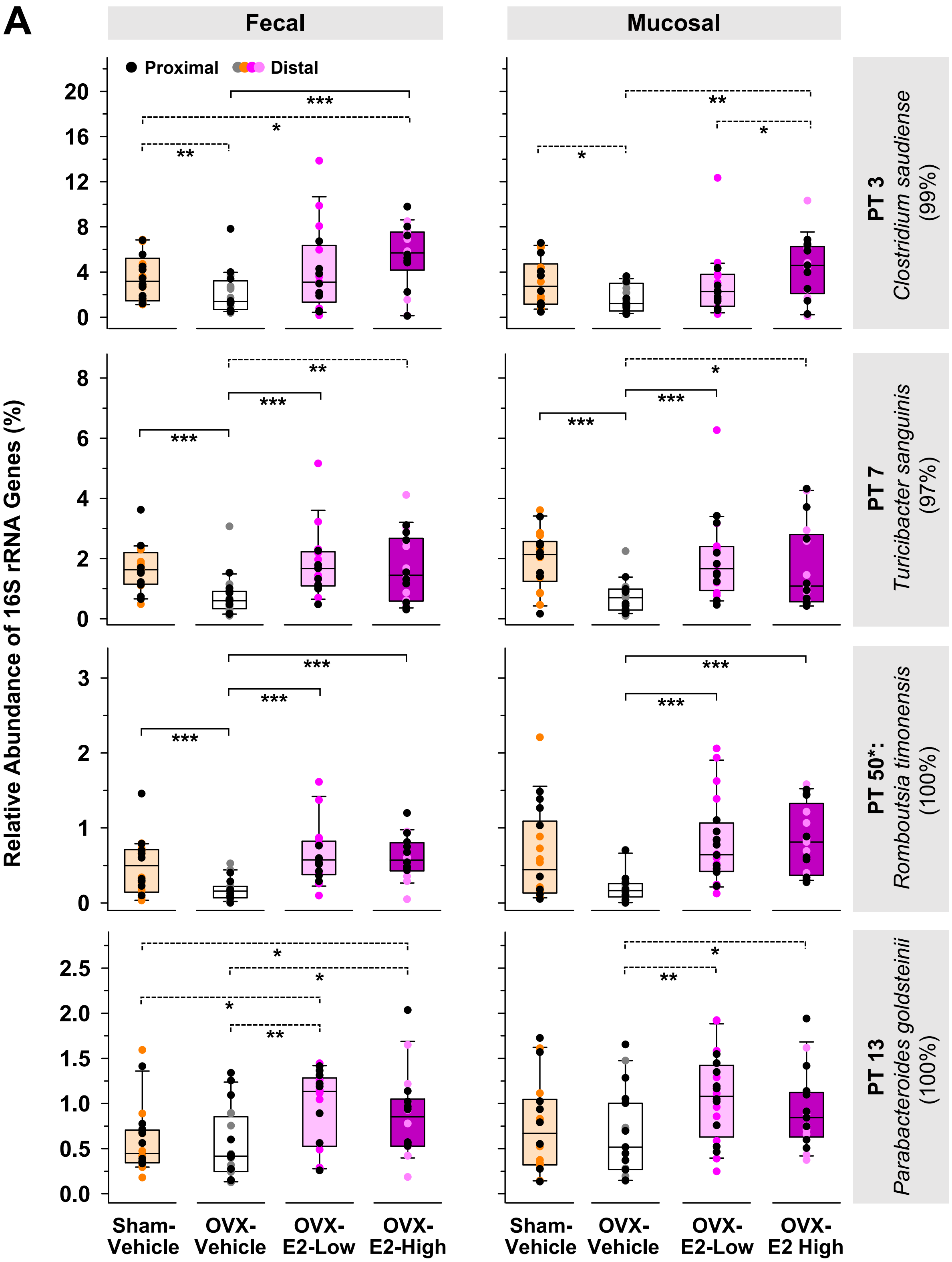
A
Fecal
Mucosal
Proximal
Distal
Relative Abundance of 16S rRNA Genes (%)
PT 3
Clostridium saudiense
(99%)
PT 7
Turicibacter sanguinis
(97%)
PT 50*:
Romboutsia timonensis
(100%)
PT 13
Parabacteroides goldsteinii
(100%)
Sham-Vehicle
OVX-Vehicle
OVX-E2-Low
OVX-E2-High
Sham-Vehicle
OVX-Vehicle
OVX-E2-Low
OVX-E2 High

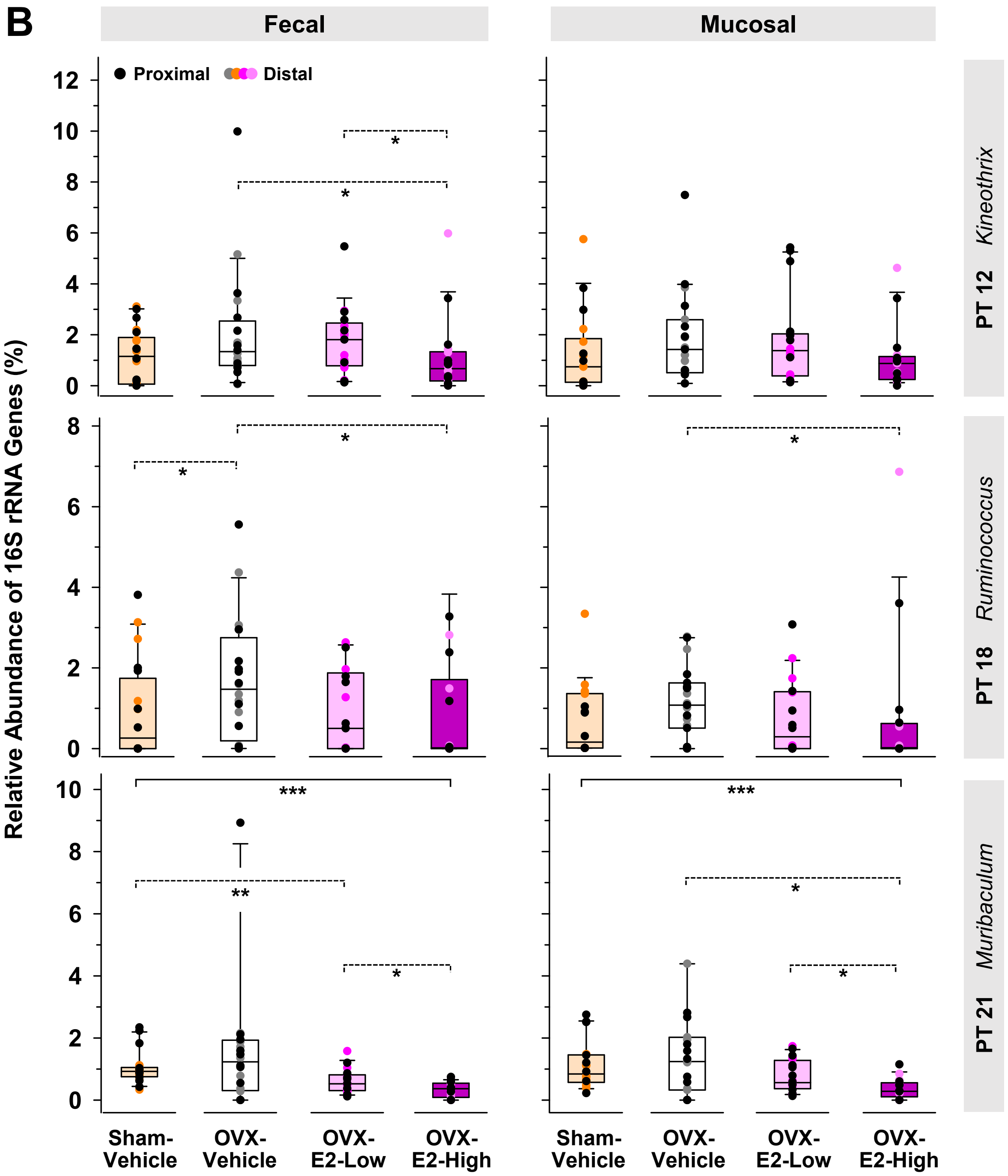

Figure S8. Intestinal phylotypes and relatives at the (A) species-level or genus-level (B) that displayed a significant difference (higher or lower relative abundances) in OVX-E2-High rats than in OVX-Vehicle rats. Statistical analysis (LEfSe) was only completed for phylotypes that had a ≥ 2% relative abundance in at least one sample. A: Percent in parenthesis indicate the sequence identity obtained from Blastn (NCBI). PT50*, Relative 16S rRNA gene abundance of PT 50 represents the sum of the relative abundance of phylotype PT 50 and PT 59 (Sequences: one nucleotide mismatch, 99.58% similarity). The asterisks indicate significant differences (*, $p \leq 0.05$; **, $p \leq 0.01$; ***, $p \leq 0.001$). Solid lines between groups indicate significant q values ($q \leq 0.05$). Dashed lines between groups indicate non-significant q values. Group abbreviations (e.g., Sham-Vehicle) are described in Table 1. Accession numbers: PT 3, LR898070; PT 7, LR898080; PT 50, LR898135; PT 59, LR898147; PT 13, LR898091; PT 12, LR898079; PT 18, LR898083; PT 21, LR898082. Error bars indicate standard deviations.

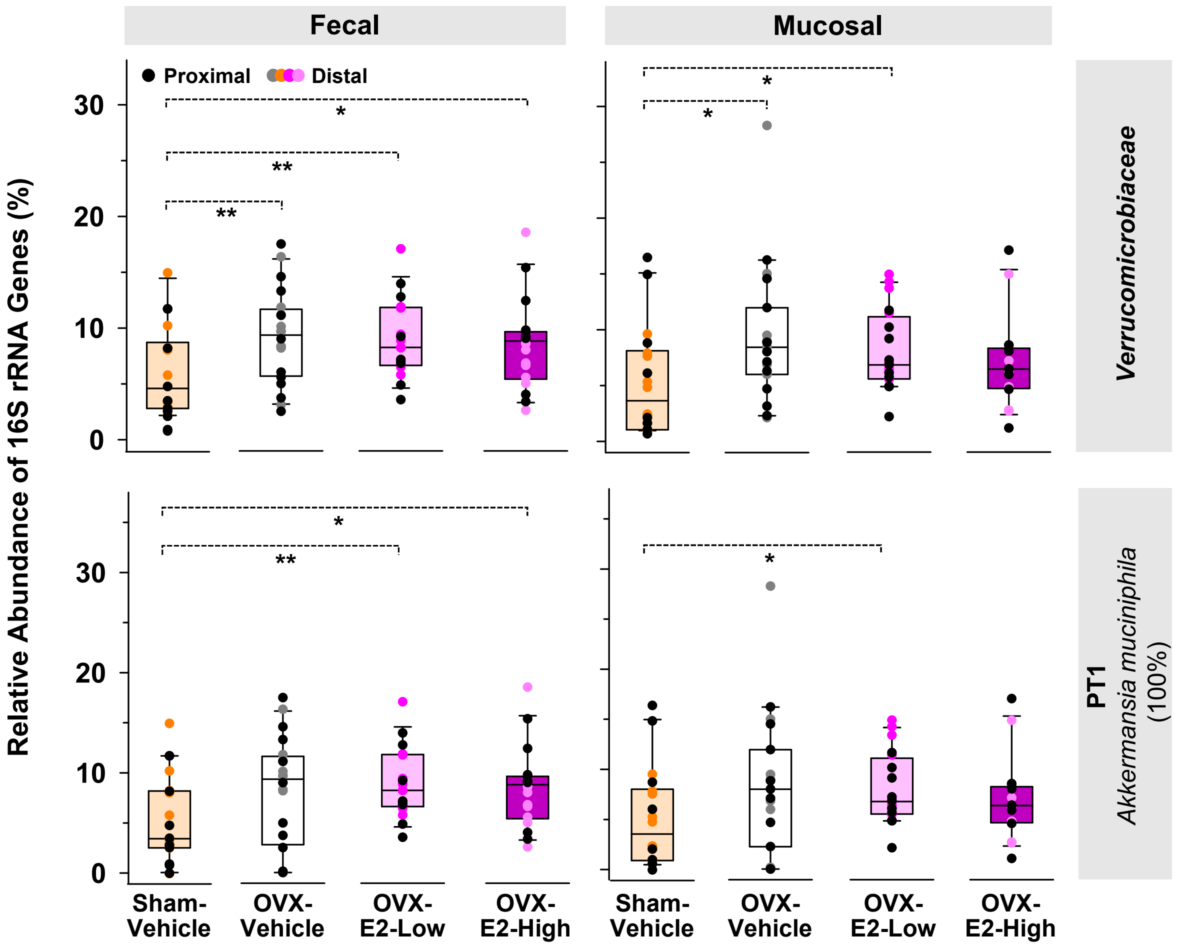

Figure S9. Relative abundance of *Verrucomicrobiaceae* and phylotype PT 1 (closely related to *Akkermansia muciniphila*). The asterisks indicate significant differences (*, p ≤ 0.05; **, p ≤ 0.01). Solid lines between groups indicate significant q values (q ≤ 0.05). Dashed lines between groups indicate non-significant q values. Group abbreviations (e.g., Sham-Vehicle) are described in Table 1. Error bars indicate standard deviations.

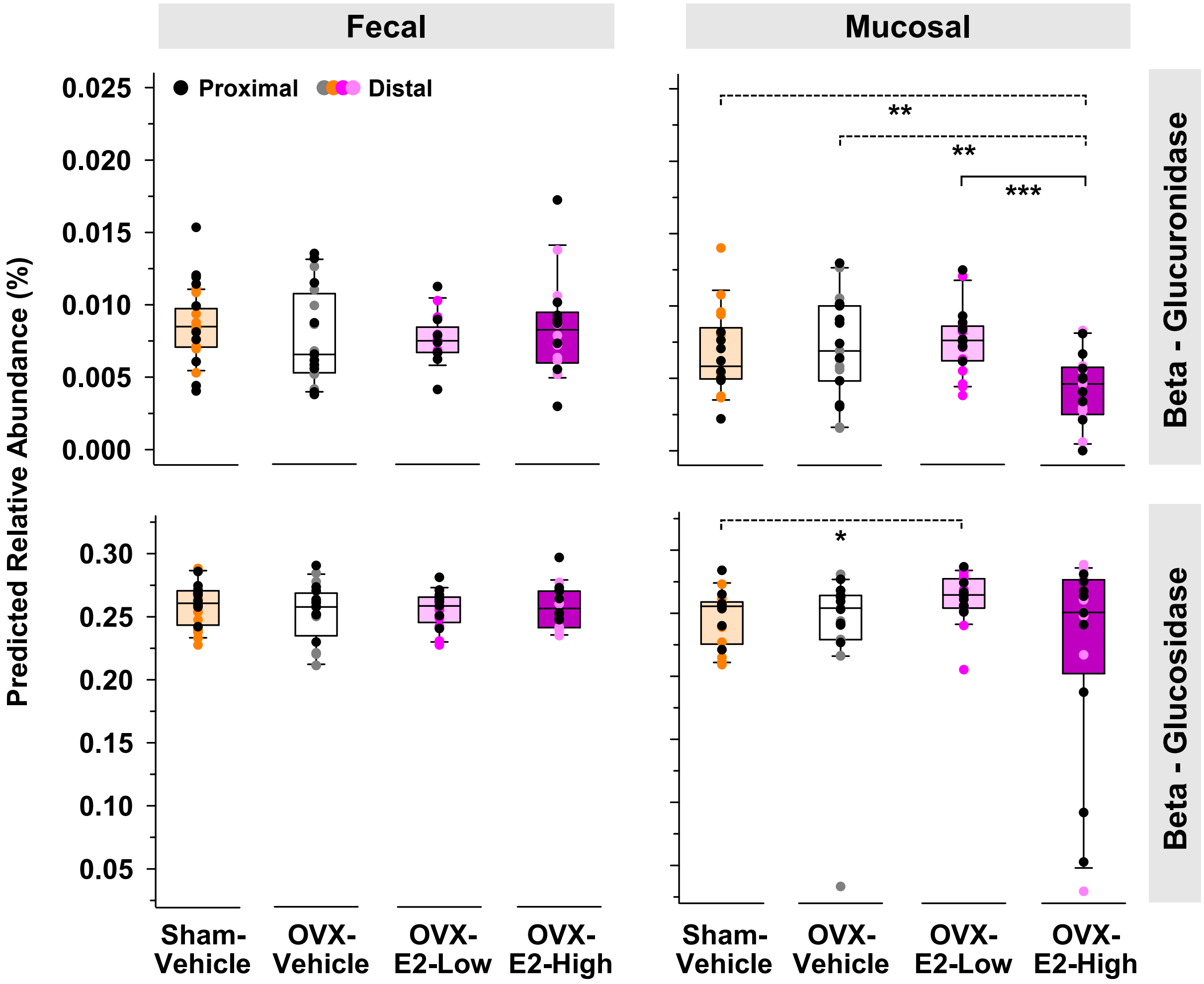

Figure S10. Predicted relative abundance of Beta-Glucuronidase and Beta-Glucosidase. The asterisks indicate significant differences (*, p ≤ 0.05; **, p ≤ 0.01; ***, p ≤ 0.001). Solid lines between groups indicate significant q values (q ≤ 0.05). Dashed lines between groups indicate non-significant q values. Group abbreviations (e.g., Sham-Vehicle) are described in Table 1. Error bars indicate standard deviations.

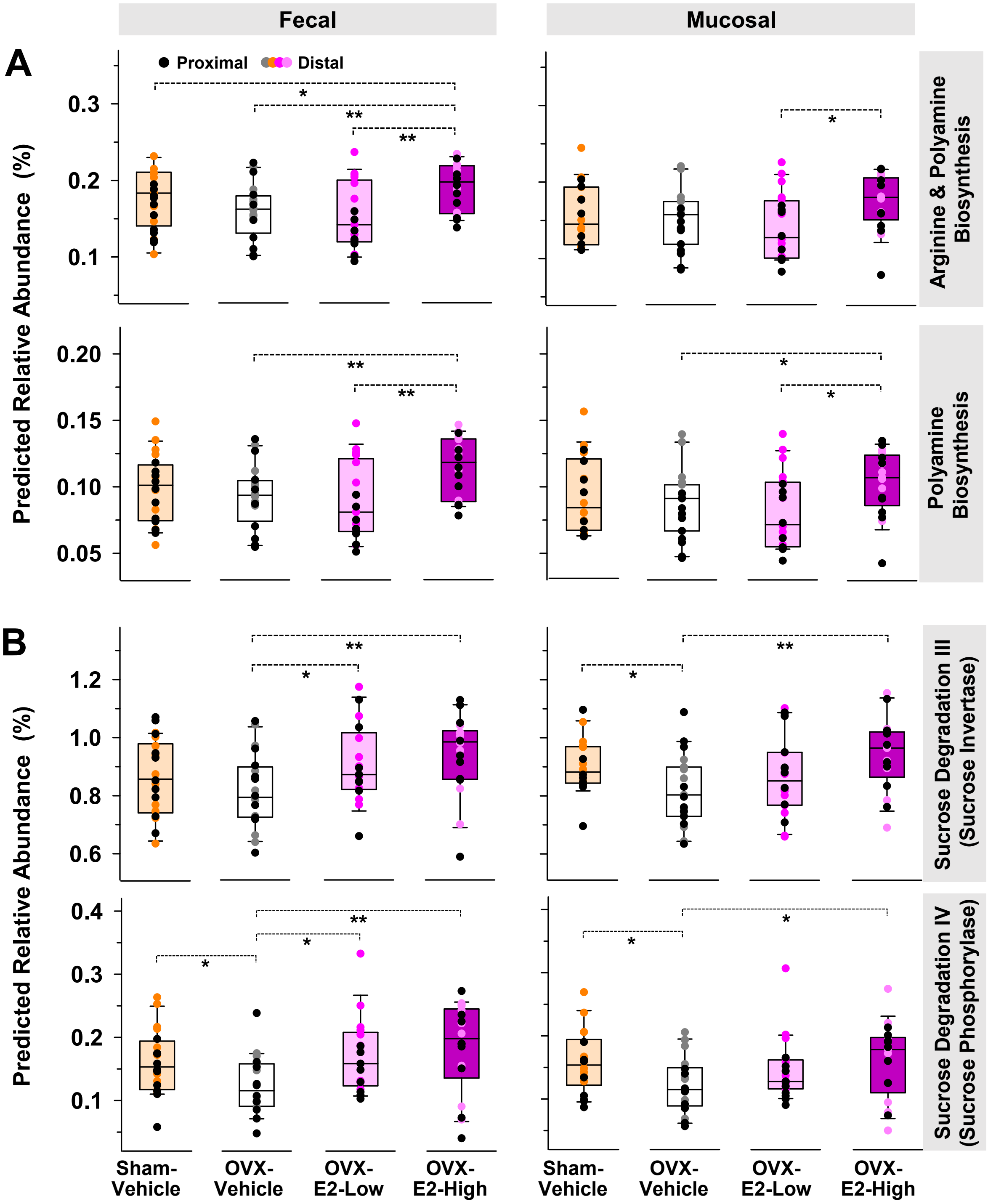

Figure S11. Predicted relative abundance of (A) two polyamide biosynthesis pathways and (B) two sucrose degradation enzymes.(ARG+POLYAMINE-SYN and POLYAMSYN-PWY). The asterisks indicate significant differences (*, p ≤ 0.05; **, p ≤ 0.01). Solid lines between groups indicate significant q values (q ≤ 0.05). Dashed lines between groups indicate non-significant q values. Group abbreviations (e.g., Sham-Vehicle) are described in Table 1. Error bars indicate standard deviations.

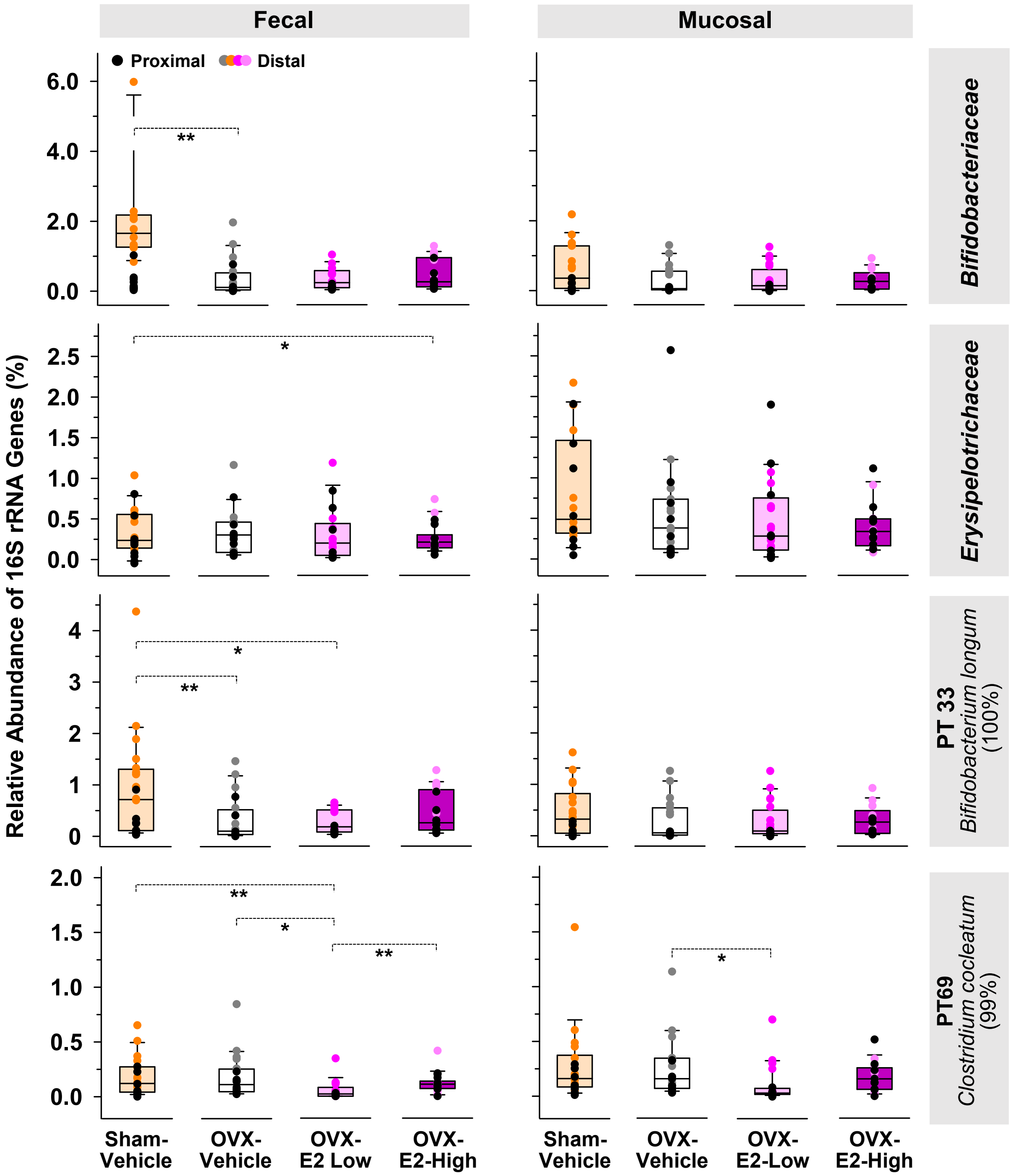

Figure S12. Relative 16S rRNA gene abundance of *Bifidobacteriaceae* and *Erysipelotrichaceae* and associated phylotypes closely related to *B. longum* and *C. cocleatum.* The asterisks indicate significant differences (*, p ≤ 0.05; **, p ≤ 0.01). Solid lines between groups indicate significant q values (q ≤ 0.05). Dashed lines between groups indicate non-significant q values. Group abbreviations (e.g., Sham-Vehicle) are described in Table 1. Error bars indicate standard deviations.

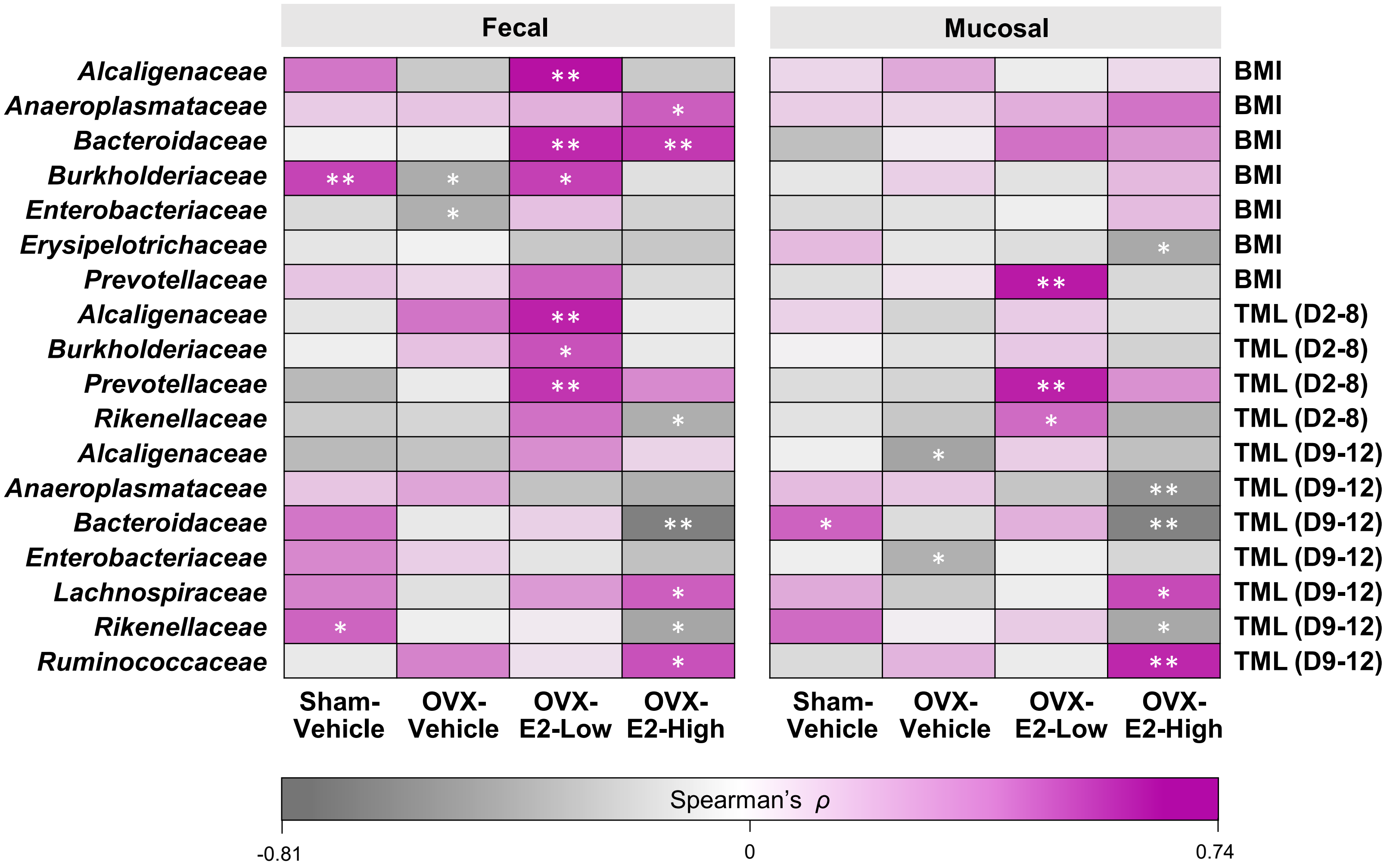

Figure S13. Heatmap of positive and negative correlations for each treatment group between cognitive data and BMI at the end of experiment, and microbial families that were not significantly affected by different E2 doses. Graph is only done with families that had a ≥ 2% relative abundance in at least one sample and displayed a significant correlation ($p \leq 0.05$) in at least one group. The asterisks indicate significant differences (*, $p \leq 0.05$; **, $p \leq 0.01$) and the shades indicate the degree of correlation. Group abbreviations (e.g., Sham-Vehicle) are described in Table 1.

Table S1. Total amount of short chain fatty acids (SCFAs) detected in the proximal or distal fecal samples of each rat.[a]

Total SCFAs (mM)											
**OVX-Vehicle**			**Sham-Vehicle**			**OVX-E2-Low**			**OVX-E2-High**		
**Subject No.** [b]	**Distal**	**Proximal**	**Subject No.** [b]	**Distal**	**Proximal**	**Subject No.** [b]	**Distal**	**Proximal**	**Subject No.** [b]	**Distal**	**Proximal**
**3**	599	400	**1**	250	1242	**5**	461	1620	**7**	9.0	1025
**4**	399	323	**2**	468	969	**6**	337	188	**8**	9.6	578
**9**	329	842	**17**	50	37	**13**	41	22	**11**	16	43
**10**	584	549	**18**	77	69	**14**	19	14	**12**	23	16
**15**	36	96	**25**	21	6.4	**19**	56	82	**23**	25	14
**16**	66	94	**26**	42	16	**20**	60	89	**24**	22	51
**21**	62	133	**29**	8.4	0.0	**27**	28	37	**35**	20	14
**22**	128	27	**30**	33	11	**28**	5.6	9.7	**36**	63	33
**37**	60	106	**33**	5.3	10	**31**	11	14	**39**	110	62
**38**	27	154	**34**	6.7	5.4	**32**	3.3	5.8	**-**	-	-

[a] Group abbreviations (e.g., Sham-Vehicle) are described in Table 1.

[b] Every subject with an even number was housed together with a subject with a following uneven number (e.g., 1 and 2, 5 and 6, 23 and 24).

Table S2. Total and relative abundance of the short chain fatty acids detected in each group in fecal samples.[a]

		SCFAs (mM)[b]									
		Formate		Acetate		Propionate		Isobutyrate		Butyrate	
		Total	Relative	Total	Relative	Total	Relative	Total	Relative	Total	Relative
**Sham-Vehicle**	Distal	48 ± 73	44 ± 28	22 ± 47	9.0 ± 12	11 ± 22	8.7 ± 6.4	1.6 ± 1.7	4.8 ± 6.2	2.1 ± 2.7	8.2 ± 16
	Proximal	131 ± 228	52 ± 25	67 ± 132	13 ± 14	35 ± 69	15 ± 15	4.4 ± 7.4	4.5 ± 4.4	8.2 ± 23	2.7 ± 6.3
**OVX-Vehicle**	Distal	117 ± 96	63 ± 18	58 ± 69	18 ± 14	32 ± 40	9.7 ± 6.1	2.5 ± 1.9	1.7 ± 1.5	3.9 ± 10	1.2 ± 2.0
	Proximal	153 ± 133	60 ± 19	60 ± 67	18 ± 12	33 ± 34	10 ± 6.4	3.0 ± 1.8	1.7 ± 1.2	6.7 ± 12	2.5 ± 3.7
**OVX-E2-Low**	Distal	51 ± 79	41 ± 22	25 ± 44	14 ± 17	13 ± 21	17 ± 14	1.6 ± 1.2	5.3 ± 6.1	6.0 ± 17	4.9 ± 10
	Proximal	100 ± 273	47 ± 22	54 ± 128	14 ± 18	27 ± 63	13 ± 11	4.2 ± 7.9	6.7 ± 12	2.1 ± 0.9	7.8 ± 5.6
**OVX-E2-High**	Distal	15 ± 23	33 ± 17	10 ± 9.9	32 ± 23	3.7 ± 4.2	11 ± 11	1.4 ± 1.0	7.0 ± 5.0	0.7 ± 1.0	3.8 ± 6.1
	Proximal	100 ± 173	47 ± 21	54 ± 104	14 ± 15	27 ± 50	13 ± 12	4.2 ± 4.3	6.7 ± 5.5	2.1 ± 3.0	7.8 ± 11
		Isovalerate		Valerate		Isocapronate		Capronate		Heptanoate	
		Total	Relative	Total	Relative	Total	Relative	Total	Relative	Total	Relative
**Sham-Vehicle**	Distal	2.1 ± 4.0	2.8 ± 4.4	2.2 ± 3.2	6.2 ± 8.2	3.5 ± 6.1	7.1 ± 8.0	2.5 ± 5.4	6.8 ± 14	1.1 ± 3.4	2.5 ± 8
	Proximal	7.1 ± 14	2.3 ± 3.5	5.1 ± 9.0	5.8 ± 4.8	4.3 ± 7.6	4.2 ± 4.4	0.0 ± 0.0	0.0 ± 0.0	0.0 ± 0.0	0.0 ± 0.0
**OVX-Vehicle**	Distal	5.2 ± 8.8	1.0 ± 1.5	4.5 ± 4.8	2.3 ± 1.0	6.3 ± 7.9	2.5 ± 1.8	0.0 ± 0.0	0.0 ± 0.0	0.0 ± 0.0	0.0 ± 0.0
	Proximal	5.3 ± 5.7	1.9 ± 1.2	5.3 ± 4.6	2.8 ± 2.8	5.3 ± 8.6	2.2 ± 3.0	0.8 ± 2.5	0.2 ± 0.6	0.0 ± 0.0	0.0 ± 0.0
**OVX-E2-Low**	Distal	1.1 ± 2.2	1.8 ± 3.7	1.7 ± 1.7	7.5 ± 8.4	1.7 ± 2.1	6.2 ± 9.2	0.0 ± 0.0	0.0 ± 0.0	0.4 ± 1.4	1.6 ± 5.0
	Proximal	5.1 ± 11	3.9 ± 16	5.0 ± 8.8	5.2 ± 5.3	4.2 ± 8.3	2.8 ± 2.2	3.0 ± 0.0	0.3 ± 0.0	0.0 ± 0.0	0.0 ± 0.0
**OVX-E2-High**	Distal	1.2 ± 2.9	7.2 ± 19	0.9 ± 0.6	3.9 ± 3.9	0.5 ± 0.7	1.9 ± 2.6	0.0 ± 0.0	0.0 ± 0.0	0.0 ± 0.0	0.0 ± 0.0
	Proximal	5.1 ± 7.7	3.9 ± 2.8	5.0 ± 7.0	5.2 ± 4.3	4.2 ± 6.3	2.8 ± 2.3	3.0 ± 7.0	0.3 ± 0.7	0.0 ± 0.0	0.0 ± 0.0

[a] Group abbreviations (e.g., Sham-Vehicle) are described in Table 1.

[b] Values are the arithmetic average of at least 8 replicate analyses (± standard deviation).

Table S3. Relative 16S rRNA abundance of *Bifidobacteriaceae* and *B. longum* (PT33) in fecal and mucosal samples, each obtained from distal and proximal gut locations.[a]

	Fecal				Mucosal			
Bifidobacteriaceae	**Sham-Vehicle**	**OVX-Vehicle**	**OVX-E2-Low**	**OVX-E2-High**	**Sham-Vehicle**	**OVX-Vehicle**	**OVX-E2-Low**	**OVX-E2-High**
Distal	2.0 ± 1.5	0.6 ± 0.7	0.6 ± 0.3	0.6 ± 0.5	1.1 ± 0.6	0.6 ± 0.4	0.6 ± 0.4	0.4 ± 0.3
Proximal	0.3 ± 0.3	0.1 ± 0.2	0.1 ± 0.1	0.3 ± 0.3	0.1 ± 0.1	0.0 ± 0.0	0.1 ± 0.1	0.2 ± 0.1
***B. longum* (PT33)**								
Distal	1.6 ± 1.1	0.5 ± 0.5	0.5 ± 0.2	0.6 ± 0.5	0.8 ± 0.5	0.6 ± 0.4	0.5 ± 0.4	0.4 ± 0.3
Proximal	0.2 ± 0.3	0.1 ± 0.2	0.1 ± 0.1	0.3 ± 0.3	0.1 ± 0.1	0.0 ± 0.0	0.1 ± 0.0	0.1 ± 0.1

[a] Group abbreviations (e.g., Sham-Vehicle) are described in Table 1.

Table S4. Families that had a ≥ 2% relative 16S rRNA gene abundances in at least one of the samples.[a]

Phyla	Family		Sham-Vehicle		OVX-Vehicle		OVX-E2-Low		OVX-E2-High	
			Distal	Proximal	Distal	Proximal	Distal	Proximal	Distal	Proximal
Firmicutes	***Ruminococcaceae***	Fecal	21 + 5.1	16 + 3.8	19 + 4.9	16 + 4.3	15 + 5.6	11 + 5.4	18 + 6.2	14 + 4.2
		Mucosal	20 + 3.3	12 + 5.7	18 + 8.4	15 + 2.8	17 + 3.0	13 + 2.5	15 + 8.5	14 + 5.3
	Lachnospiraceae	Fecal	7.8 + 2.8	7.5 + 2.8	8.7 + 4.5	8.3 + 4.2	8.5 + 3.9	8.6 + 5.2	9.4 + 3.3	7.5 + 3.1
		Mucosal	12 + 6.2	6.5 + 4.3	9.4 + 5.5	8.8 + 3.8	9.7 + 4.0	12 + 4.3	9.4 + 6.1	11 + 3.6
	Clostridiaceae	Fecal	4.2 + 2.3	3.9 + 2.1	2.4 + 1.4	2.7 + 2.6	5.3 + 4.6	2.6 + 2.5	6.5 + 3.4	6.0 + 3.3
		Mucosal	3.5 + 2.4	2.8 + 2.6	2.3 + 1.4	1.7 + 1.3	3.8 + 3.5	2.5 + 1.4	4.6 + 3.8	4.8 + 2.7
	**Unassigned**	Fecal	17 + 3.5	21 + 8.1	19 + 6.8	20 + 4.5	16 + 6.9	18 + 9.9	17 + 3.0	18 + 7.2
	Clostridiales	Mucosal	24 + 6.0	18 + 15	18 + 8.0	24 + 7.6	18 + 6.2	28 + 9.9	19 + 8.4	24 + 9.6
	Lactobacillaceae	Fecal	1.7 + 1.0	1.1 + 1.0	1.9 + 1.4	1.4 + 1.1	1.2 + 0.8	0.7 + 0.3	1.1 + 0.4	1.3 + 0.6
		Mucosal	1.3 + 0.7	0.7 + 0.7	1.9 + 1.4	0.9 + 0.6	1.3 + 0.9	0.8 + 0.3	1.1 + 0.8	1.2 + 0.8
	Turici-	Fecal	1.7 + 0.6	1.6 + 0.9	0.8 + 0.9	0.7 + 0.4	2.0 + 1.4	1.1 + 0.7	1.9 + 1.3	1.6 + 1.1
	bacteraceae	Mucosal	2.0 + 1.0	1.6 + 1.2	0.8 + 0.7	0.6 + 0.4	2.3 + 1.6	1.7 + 1.1	1.6 + 1.5	1.7 + 1.6
	Peptostrepto-	Fecal	0.4 + 0.3	0.5 + 0.4	0.2 + 0.2	0.2 + 0.1	0.7 + 0.5	0.4 + 0.3	0.5 + 0.3	0.7 + 0.3
	coccaceae	Mucosal	0.6 + 0.6	0.6 + 0.6	0.2 + 0.2	0.2 + 0.2	0.9 + 0.7	0.7 + 0.3	0.8 + 0.5	0.8 + 0.5
	Erysipelo-	Fecal	0.5 + 0.3	0.3 + 0.3	0.4 + 0.3	0.3 + 0.2	0.3 + 0.3	0.2 + 0.3	0.3 + 0.2	0.2 + 0.1
	trichaceae	Mucosal	0.9 + 0.7	0.7 + 0.7	0.5 + 0.4	0.5 + 0.8	0.5 + 0.3	0.5 + 0.6	0.3 + 0.3	0.4 + 0.3
	Peptococcaceae	Fecal	0.3 + 0.2	0.6 + 0.3	0.5 + 0.2	0.7 + 0.4	0.3 + 0.2	0.6 + 0.7	0.4 + 0.2	0.8 + 0.6
		Mucosal	0.4 + 0.2	0.6 + 0.5	0.3 + 0.2	0.9 + 0.6	0.4 + 0.1	0.7 + 0.5	0.5 + 0.4	0.7 + 0.5
	**Other Firmicutes**	Fecal	0.4 + 0.2	0.2 + 0.1	0.3 + 0.1	0.2 + 0.1	0.3 + 0.2	0.3 + 0.4	0.3 + 0.1	0.2 + 0.1
		Mucosal	0.4 + 0.2	0.2 + 0.1	0.3 + 0.2	0.2 + 0.0	0.5 + 0.2	0.2 + 0.1	0.3 + 0.2	0.3 + 0.1

Phyla	Family		Sham-Vehicle		OVX-Vehicle		OVX-E- Low		OVX-E2-High	
			Distal	Proximal	Distal	Proximal	Distal	Proximal	Distal	Proximal
**Bacteroidetes**	***Prevotellaceae***	Fecal	2.9 + 3.7	5.1 + 2.6	4.5 + 4.7	6.6 + 5.4	3.6 + 3.2	6.9 + 6.8	3.1 + 3.0	5.6 + 3.9
		Mucosal	3.0 + 3.3	5.3 + 4.6	3.0 + 3.6	6.8 + 5.6	4.0 + 3.0	6.7 + 5.0	4.4 + 5.8	4.1 + 2.9
	Bacteroidaceae	Fecal	2.1 + 2.2	2.7 + 2.1	2.2 + 1.8	2.9 + 1.8	1.1 + 0.6	1.6 + 0.8	1.7 + 1.0	2.1 + 1.3
		Mucosal	2.0 + 1.8	2.6 + 2.1	1.7 + 1.7	3.1 + 2.0	1.4 + 0.7	1.7 + 0.8	1.7 + 1.2	2.3 + 1.0
	Unassigned Bacteroidales	Fecal	27 + 5.1	27 + 9.0	24 + 7.4	24 + 10	23 + 8.0	18 + 9.7	25 + 7.4	26 + 9.3
		Mucosal	20 + 5.9	20 + 12	19 + 11	20 + 7.3	24 + 7.4	19 + 6.2	20 + 11	20 + 5.8
	Rikenellaceae	Fecal	0.6 + 0.6	1.0 + 0.8	1.1 + 0.7	1.6 + 1.0	1.1 + 0.9	1.9 + 1.6	1.6 + 1.3	2.1 + 1.7
		Mucosal	0.8 + 0.9	1.0 + 1.3	0.8 + 0.7	2.6 + 2.2	1.3 + 1.0	2.0 + 1.2	1.1 + 1.2	1.4 + 0.8
	Porphyromona-daceae	Fecal	0.6 + 0.4	0.6 + 0.3	0.5 + 0.3	0.6 + 0.5	0.8 + 0.5	1.0 + 0.6	0.8 + 0.5	0.9 + 0.5
		Mucosal	0.6 + 0.5	0.7 + 0.6	0.5 + 0.4	0.7 + 0.5	1.1 + 0.6	1.1 + 0.4	0.7 + 0.5	1.0 + 0.4
	Barnesiellaceae	Fecal	0.0 + 0.0	0.0 + 0.0	0.0 + 0.0	0.0 + 0.0	0.0 + 0.0	0.0 + 0.0	0.0 + 0.0	0.0 + 0.0
		Mucosal	0.0 + 0.0	0.0 + 0.0	0.0 + 0.0	0.0 + 0.0	0.0 + 0.0	0.0 + 0.0	0.0 + 0.0	0.0 + 0.0
	**Other *Bacteroidetes***	Fecal	0.0 + 0.0	0.1 + 0.4	0.0 + 0.0	0.0 + 0.0	0.0 + 0.0	0.1 + 0.2	0.0 + 0.0	0.0 + 0.0
		Mucosal	0.0 + 0.0	0.0 + 0.0	0.0 + 0.0	0.0 + 0.0	0.0 + 0.0	0.0 + 0.0	0.1 + 0.3	0.0 + 0.0
**Tenericutes**	**Unassigned *Mollicutes***	Fecal	1.4 + 0.9	0.9 + 0.6	2.3 + 2.4	1.8 + 1.2	1.0 + 0.9	0.5 + 0.4	1.5 + 0.6	1.3 + 0.8
		Mucosal	1.0 + 0.6	0.7 + 0.7	2.1 + 2.0	1.6 + 1.1	1.1 + 1.0	0.9 + 0.8	1.1 + 0.6	1.0 + 0.4
	Anaero-plasmataceae	Fecal	0.1 + 0.2	0.5 + 0.9	0.1 + 0.2	0.4 + 0.9	0.1 + 0.3	0.2 + 0.4	0.4 + 1.0	0.5 + 1.0
		Mucosal	0.4 + 0.8	0.3 + 0.6	0.1 + 0.2	0.4 + 0.6	0.3 + 0.7	0.4 + 0.6	0.3 + 0.7	0.3 + 0.5
	**Other Tenericutes**	Fecal	0.0 + 0.0	0.1 + 0.1	0.0 + 0.0	0.0 + 0.0	0.0 + 0.1	0.0 + 0.0	0.0 + 0.1	0.0 + 0.1
		Mucosal	0.1 + 0.1	0.2 + 0.3	0.0 + 0.0	0.0 + 0.1	0.0 + 0.1	0.0 + 0.1	0.0 + 0.1	0.1 + 0.1

			Sham-Vehicle		OVX-Vehicle		OVX-E2-Low		OVX-E2-High	
**Phyla**	**Family**		Distal	Proximal	Distal	Proximal	Distal	Proximal	Distal	Proximal
**Proteobacteria**	***Burkholderiaceae***	Fecal	0.4 + 0.9	0.8 + 0.9	0.5 + 0.8	0.4 + 0.5	6.2 + 18	12 + 25	0.7 + 0.9	0.2 + 0.6
		Mucosal	0.5 + 0.9	12 + 24	0.4 + 0.6	0.5 + 0.4	0.9 + 0.6	0.3 + 0.5	7.1 + 20	1.1 + 1.0
	Alcaligenaceae	Fecal	0.3 + 0.3	0.6 + 0.6	0.4 + 0.4	0.4 + 0.3	2.2 + 6.2	5.6 + 11	0.5 + 0.4	0.3 + 0.3
		Mucosal	0.4 + 0.5	4.1 + 7.9	0.3 + 0.4	0.5 + 0.4	0.8 + 0.5	0.3 + 0.2	3.2 + 8.5	0.7 + 0.5
	**Unassigned**	Fecal	0.1 + 0.1	0.2 + 0.3	0.2 + 0.2	0.4 + 0.6	0.1 + 0.1	0.2 + 0.4	0 + 0.1	0.1 + 0.1
	**α-Proteobacteria**	Mucosal	0.1 + 0.2	0.2 + 0.3	0.1 + 0.1	0.6 + 0.9	0.1 + 0.2	0.2 + 0.3	0.1 + 0.1	0.1 + 0.1
	Comamona-	Fecal	0.0 + 0.0	0.0 + 0.1	0.0 + 0.0	0.0 + 0.0	1.0 + 3.1	0.1 + 0.3	0.0 + 0.1	0.0 + 0.0
	daceae	Mucosal	0.0 + 0.0	0.3 + 0.8	0.0 + 0.0	0.0 + 0.0	0.0 + 0.1	0.1 + 0.1	0.3 + 0.7	0.0 + 0.0
	Halomona-	Fecal	0.0 + 0.0	0.0 + 0.0	0.0 + 0.1	0.0 + 0.0	0.0 + 0.0	0.2 + 0.5	0.0 + 0.0	0.0 + 0.1
	daceae	Mucosal	0.0 + 0.0	0.4 + 1.3	0.0 + 0.0	0.1 + 0.1	0.0 + 0.0	0.0 + 0.0	0.1 + 0.3	0.0 + 0.1
	Bradyrhizo-	Fecal	0.0 + 0.1	0.0 + 0.0	0.0 + 0.0	0.0 + 0.0	0.1 + 0.3	0.2 + 0.4	0.0 + 0.0	0.0 + 0.0
	biaceae	Mucosal	0.0 + 0.0	0.5 + 1.3	0.0 + 0.0	0.0 + 0.0	0.0 + 0.0	0.0 + 0.0	0.0 + 0.0	0.0 + 0.0
	Oxalo-	Fecal	0.0 + 0.0	0.0 + 0.0	0.0 + 0.0	0.0 + 0.0	0.1 + 0.2	0.4 + 0.7	0.0 + 0.1	0.0 + 0.0
	bacteraceae	Mucosal	0.0 + 0.0	0.2 + 0.7	0.0 + 0.0	0.0 + 0.0	0.0 + 0.0	0.0 + 0.0	0.0 + 0.0	0.0 + 0.0
	Entero-	Fecal	0.0 + 0.0	0.0 + 0.0	0.0 + 0.0	0.0 + 0.0	0.0 + 0.0	0.0 + 0.0	0.0 + 0.0	0.0 + 0.0
	bacteriaceae	Tissue	0.0 + 0.1	0.3 + 0.8	0.0 + 0.1	0.0 + 0.0	0.0 + 0.0	0.0 + 0.0	0.0 + 0.1	0.0 + 0.1
	**Other**	Fecal	0.0 + 0.0	0.1 + 0.1	0.0 + 0.0	0.1 + 0.1	0.0 + 0.1	0.1 + 0.2	0.0 + 0.0	0.0 + 0.0
	**Proteobacteria**	Tissue	0.1 + 0.1	0.1 + 0.2	0.2 + 0.5	0.1 + 0.1	0.1 + 0.1	0.2 + 0.6	0.1 + 0.2	0.0 + 0.0

Phyla	Family		Sham-Vehicle Distal	Sham-Vehicle Proximal	OVX-Vehicle Distal	OVX-Vehicle Proximal	OVX-E2-Low Distal	OVX-E2-Low Proximal	OVX-E2-High Distal	OVX-E2-High Proximal
Actinobacteria	***Bifido-bacteriaceae***	Fecal	2.0 + 1.5	0.3 + 0.3	0.6 + 0.7	0.1 + 0.2	0.5 + 0.3	0.1 + 0.1	0.7 + 0.5	0.3 + 0.3
		Mucosal	1.1 + 0.6	0.2 + 0.3	0.5 + 0.4	0.0 + 0.0	0.6 + 0.4	0.1 + 0.1	0.4 + 0.3	0.1 + 0.1
	Coryne-bacteriaceae	Fecal	0.0 + 0.0	0.0 + 0.0	0.0 + 0.0	0.0 + 0.0	0.0 + 0.0	0.0 + 0.0	0.0 + 0.0	0.0 + 0.0
		Mucosal	0.0 + 0.0	0.0 + 0.0	0.0 + 0.0	0.0 + 0.0	0.0 + 0.0	0.0 + 0.0	0.3 + 1.0	0.0 + 0.0
	**Other Actinobacteria**	Fecal	0.2 + 0.1	0.1 + 0.1	0.2 + 0.1	0.1 + 0.1	0.1 + 0.1	0.0 + 0.1	0.1 + 0.1	0.0 + 0.0
		Mucosal	0.2 + 0.1	0.1 + 0.1	0.2 + 0.2	0.0 + 0.0	0.2 + 0.1	0.0 + 0.0	0.1 + 0.1	0.1 + 0.1
**Verrucomicrobia (*Verrucomicrobiaceae*)**		Fecal	6.2 + 4.2	5 + 4.2	9.7 + 3.3	8.8 + 5.1	9.0 + 4.0	6.5 + 4.7	7.9 + 4.5	9.1 + 3.7
		Mucosal	4.3 + 3.2	5.2 + 6.2	19 + 29	8.3 + 4.7	9.1 + 4.0	6.9 + 2.8	5.5 + 4.2	7.4 + 4.3
**Cyanobacteria**		Fecal	0.1 + 0.2	1.5 + 2.0	0.2 + 0.3	1.0 + 1.5	0.1 + 0.1	0.4 + 0.6	0.2 + 0.4	0.8 + 0.6
		Mucosal	0.0 + 0.0	0.0 + 0.0	0.0 + 0.0	0.0 + 0.0	0.0 + 0.0	0.0 + 0.0	0.0 + 0.0	0.0 + 0.0
**Others**		Fecal	0.1 + 0.1	0.3 + 0.3	0.1 + 0.1	0.1 + 0.1	0.1 + 0.1	0.3 + 0.3	0.1 + 0.1	0.2 + 0.2
		Mucosal	0.3 + 0.3	0.3 + 0.3	0.3 + 0.3	0.6 + 0.8	0.3 + 0.5	0.4 + 0.2	0.3 + 0.2	0.5 + 0.4

[a] Group abbreviations (e.g., Sham-Vehicle) are described in Table 1. Values are the arithmetic average of at least 8 replicate analyses (± standard deviation).
